# Detection of selection signatures for response to Aleutian mink disease virus infection in American mink

**DOI:** 10.1038/s41598-021-82522-8

**Published:** 2021-02-03

**Authors:** Karim Karimi, A. Hossain Farid, Sean Myles, Younes Miar

**Affiliations:** 1 Department of Animal Science and Aquaculture, Dalhousie University, Truro, NS Canada; 2 Department of Plant, Food, and Environmental Sciences, Dalhousie University, Truro, NS Canada

**Keywords:** Agricultural genetics, Animal breeding, Genetic markers, Genome

## Abstract

Aleutian disease (AD) is the most significant health issue for farmed American mink. The objective of this study was to identify the genomic regions subjected to selection for response to infection with Aleutian mink disease virus (AMDV) in American mink using genotyping by sequencing (GBS) data. A total of 225 black mink were inoculated with AMDV and genotyped using a GBS assay based on the sequencing of *ApeKI*-digested libraries. Five AD-characterized phenotypes were used to assign animals to pairwise groups. Signatures of selection were detected using integrated measurement of fixation index (F_ST_) and nucleotide diversity (θπ), that were validated by haplotype-based (hap-FLK) test. The total of 99 putatively selected regions harbouring 63 genes were detected in different groups. The gene ontology revealed numerous genes related to immune response (e.g. *TRAF3IP2*, *WDR7*, *SWAP70*, *CBFB*, and *GPR65*), liver development (e.g. *SULF2*, *SRSF5*) and reproduction process (e.g. *FBXO5*, *CatSperβ*, *CATSPER4*, and *IGF2R*). The hapFLK test supported two strongly selected regions that contained five candidate genes related to immune response, virus–host interaction, reproduction and liver regeneration. This study provided the first map of putative selection signals of response to AMDV infection in American mink, bringing new insights into genomic regions controlling the AD phenotypes.

## Introduction

Aleutian disease (AD), caused by a contagious parvovirus in the genus *Amdoparvovirus*, is the most significant health issue for farmed American mink (*Neovison vison*) across the world. Infection with Aleutian mink disease virus (AMDV) stimulates immune responses to produce high levels of persistent antibodies which cannot neutralize the virus, and subsequently leads to the formation and deposition of immune complexes causing kidney damage and high mortality especially in kits^1^. In addition, one or more of other symptoms such as arteritis^2^, hepatitis^3^ and splenomegaly^4^ were reported in some infected adult mink. Furthermore, AD is generally associated with infertility, abortion, low fur quality and weight loss of infected animals^5,6^, leading to prominent economic losses in mink industry worldwide.

Although immunological tests have been used to detect and eliminate infected animals, this strategy has not been successful in complete eradication of the virus from mink farms. For instance, despite the successful use of counterimmunoelectrophoresis (CIEP) test to reduce the prevalence of infection on mink farms in the Canadian province of Nova Scotia, this strategy failed to eradicate the virus on most farms^7^. In addition, a high persistence of AMDV was observed in the breeding environments causing the reinfection of mink farms and making it difficult to control the prevalence of the disease^8,9^. Thus far, no effective vaccine or treatment is available for AD^10^. However, some non-Aleutian mink can tolerate the infection, showing normal levels of serum gamma-globulin, low anti-AMDV antibody titer, and mild or no gross or microscopic lesions characteristic of AD^11–14^. Moreover, it is well documented that some AMDV-infected mink do not succumb to the disease and live healthy lives^14–16^, suggesting that selection for tolerance to AD is a feasible approach to control AD on mink farms. Characterizing the genomic regions contributing to AD tolerance could be an effective way to develop breeding programs for increased AD resiliency on mink farms.

Development of whole-genome sequencing technologies has facilitated the identification of single nucleotide polymorphisms (SNPs) across the genome and provided a powerful tool to discover the genomic regions targeted by selection pressures in different species^17^. Genome-wide detection of selection signatures has widely been used to understand the genetic basis of domestication^18,19^, the process of adaptation to extreme environments^20,21^, and the effects of positive selection on genetic composition of economically important traits in livestock species^22,23^. However, to our knowledge, no study has been conducted in American mink to scan the genome for selective sweeps. Identification of selection signatures in American mink requires genotyping a huge number of SNP markers throughout the genome in many individuals. Genotyping by sequencing (GBS) is a cost-effective approach to discover a large number of SNPs across the genome using next generation sequencing technologies^24^. The use of this approach can provide the opportunity to identify DNA markers underlying the adaptive changes for the traits of interest in American mink.

The objective of this study was to use the genome-wide SNP markers extracted by GBS technique to reveal the genomic regions under selection in a group of black American mink inoculated with AMDV.

## Materials and methods

### Animals and sampling

Animal management and sampling protocols were performed in accordance with the standards of the Canadian Council on Animal Care (http://www.ccac.ca) after approval by the Dalhousie University Animal Care and Use Committee. Prior to inoculation, sampling and euthanasia, animals were anesthetized as previously explained^25^. Animals which were used in the current experiment were chosen from a group of 1435 black American mink which were inoculated in the Falls of 2010, 2011 and 2012. The viral inoculum was a 10% (W/V) passage 2 of AMDV prepared from the spleens of mink infected with a local strain and stored at − 80 °C as previously described^25^. Animals were intranasally inoculated under sedation with 60 µL of the viral homogenate, which corresponded to approximately 300 to 700 ID_50_, depending on the method and time of detection of infection^26^. Intranasal inoculation of sedated mink was found to be an effective method of establishing infection without destroying animals’ physical barrier^27^, thus resembling natural infection. Animals were kept in individual cages at a bio-secure facility (Aleutian Disease Research Centre) and fed a commercial dry pellet (National Feeds Inc., Maria Stern, OH, USA, https://www.manta.com/c/mr55j1c/national-feeds-inc). Animals had free access to feed and water and were monitored daily. The date and signs of dead and sick animals were recorded. Healthy animals with high reproductive performance were retained for breeding, and extra animals were killed in January and February each year.

Blood samples were collected by toenail clipping under anesthesia at least three times each year, starting at day 35 post-inoculation (pi). Blood was collected in heparinized capillary tubes for antibody testing by the CIEP, and in EDTA-coated capillary tubes for viral DNA detection by the polymerase chain reaction (PCR). By the time of conducting this experiment, 644 of the mink had been euthanized and their blood, lungs, liver, kidneys, heart and spleen samples had been collected. The lungs, kidneys and liver samples were stored in 10% formalin for histopathological evaluation. Among the inoculated animals, 392 individuals had died or terminated due to AD.

### Laboratory procedures

The presence of antibodies in the plasma was evaluated using CIEP test^12^, and antibody titers of euthanized mink were measured by CIEP using 11 two-fold serial dilutions of plasma samples (1, 1/2, 1/4, 1/8, 1/16, 1/32, 1/64, 1/128, 1/256, 1/512 and 1/1024). Antibody titer was recorded as the highest level of dilution that resulted in positive CIEP test^25^. DNA was extracted from plasma and from cell-free tissue suspensions using Dynabeads Silane viral nucleic acid extraction kit (Invitrogen, Burlington, ON). The PCR was used to test the presence of viral DNA using primer 60F and 60R as previously explained^28^. Furthermore, severity of AD symptoms was determined in the lung, kidney and liver samples by an experienced pathologist at the Pathology Laboratory, Nova Scotia Department of Agriculture (Truro, Canada). Histolopathological lesions were measured based on a scale from 0 (no lesion) to 4 (very severe lesions), as an indicator of accumulation of plasma cells in tissues^29^.

### Libraries preparation and DNA sequencing

The mink that were selected for this experiment (n = 225) were those with a wide range of responses to AMDV infection (Table 1). High quality DNA was extracted from the spleen tissue of each animal using the high-salt method^30^. Quality of DNA was checked by gel electrophoresis and DNA samples were quantified by a NanoDrop 1000 spectrophotometer (Thermo Scientific, Waltham, MA, USA). Samples with 260/280 ratio greater than 1.7 were selected to assure purity of DNA. High quality DNA was digested with the restriction enzyme *ApeKI*. Barcode adaptors along with a standard Y-adaptor were ligated to DNA fragments. DNA amplification was performed by PCR to generate three sets of 96-plex GBS libraries. Finally, DNA sequencing was performed on three lanes of the Illumina HiSeq Sequencer at the Génome Québec Innovation Centre.

**Table 1:** The phenotypic traits used to classify individuals in five pairwise groups based on the response to AMDV infection.

Group	Measurements	Positive subgroup		Negative subgroup	
		Number of individuals	Description	Number of individuals	Description
Survival	Survived or death due to sickness	20	Mink which died or terminated due to sickness and were PCR positive at day 35 pi	52	Mink were alive until euthanasia and had no lesions in the lungs, liver and kidneys
Kidney lesions	Severity of AD symptoms in kidneys	30	Inoculated mink had lesions (1 to 4 scales of severity)	142	Mink had no lesion in kidneys
Antibody titer	CIEP test of serially diluted plasma	23	Antibody titres ≤ 1/128	56	Antibody titres ≥ 1/4
Virus clearance	Virus in the spleen	128	Positive PCR test	43	Negative PCR test
Viremia-350	Virus in plasma, day 350 pi	17	Positive PCR test	156	Negative PCR test

### Sequences analysis and quality control

In total, more than 681 million of 100 bp single-end reads were generated by the Illumina HiSeq Sequencer. Unique adaptor barcodes were used to demultiplex reads into separate files using Sabre software (https://github.com/najoshi/sabre). Primers sequences, adaptor contaminations and all reads shorter than 50 bp were discarded using Cutadapt^31^. All reads were mapped to the American mink reference genome^32^ using BWA-MEM with default parameters^33^. Then, the variant calling was performed by Genome Analysis ToolKit (GATK)’s HaplotypeCaller^34^. The following metrics were implemented in GATK to filter variants with a quality by depth (QD) ˂2.0, mapping quality (MQ) < 40.0, Fisher strand (FS) > 60.0, mapping quality rank sum test <  − 12.5, and read position rank sum test <  − 8.0. Furthermore, all SNPs with a minor allele frequency (MAF) < 0.05, call rate < 0.90 and those deviating from Hardy–Weinberg equilibrium (*P* < 10^−6^) were filtered out. In addition, individuals with > 0.15 missing genotypes were discarded from the data set using VCFtools^35^. Minor allele frequency was computed for all SNPs and proportion of SNPs was determined for MAF ranges of < 0.05, 0.05 to < 0.1, 0.1 to < 0.2, 0.2 to < 0.3, 0.3 to < 0.4 and 0.4 to ≤ 0.5.

### Animals grouping

Five phenotypic parameters including antibody titer, survival or death due to AD, severity of AD symptoms in the kidneys, virus clearance (the presence or absence of AMDV DNA in the spleen) and viremia on day 350 pi (PCR on plasma) were used to divide animals into positive and negative subgroups based on the level of response to AMDV infection. Table 1 presents the five pairwise groups as well as the number of individuals in each subgroup and the criteria considered for each grouping. In addition, Supplementary Fig. S1 shows the number of individuals shared among different phenotypic groups.

### Detection of selection signatures

#### Analysis using genetic differentiation measures

In this study, several approaches were used to detect selection signatures in the genome of American mink. First, a genome scan for selection signatures was performed based on the combination of genome-wide pairwise F_ST_^36^ and nucleotide diversity (θ_π_)^37^ within each group of animals. Both statistics were calculated for each SNP and averaged along 100 kb windows with a step size of 25 kb using VCFtools. The F_ST_ values were then Z-transformed (Z(F_ST_)) and visualised using a scaffold-based circos plot^38^ for all groups. Moreover, θ_π_ ratios were computed as θ_π-Negative_/θ_π-Positive_ for all pairs of groups and were then log_2_-transformed (log_2_ (θ_π ratios_)). All of the windows including the top 1% values of both Z(F_ST_) and log_2_ (θ_π ratios_) were considered as the candidate selection regions. Since the results of differentiation methods are likely to be inaccurate for small scaffolds, only scaffolds ≥ 10 Mb (134 scaffolds including 29,914 SNPs) were used in the analyses.

#### Analysis using hapFLK test

The hapFLK test uses the local haplotype structures as well as haplotype allele frequencies and hierarchical structure of populations to reveal the signatures of selection. This approach takes into account a neutral model for SNP data and can be applied to unphased genomic data^39^. The hapFLK test was used to further validate the candidate regions identified by the previously described measures. We used the haplotype-based statistic (hapFLK) to reveal the selection signals accounting for differentiation of haplotype structures among groups. Various numbers of haplotype clusters (− K 10 to − K 40) were tested using fastPHASE software^40^ and − K 30 was selected as the best number of clusters in the hapFLK analyses. The kinship matrix and Reynolds’ genetic distance were computed by hapFLK v.1.40 for each scaffold. In addition, no outgroup was considered, and the expected maximum number of iterations was set to be 20 to fit the LD model.

#### *P*-value calculation

It was assumed that the putatively selected regions include only a small fraction of the genome^41^. Therefore, the distribution of hapFLK values can be explained by a normal distribution except for a small proportion of outliers related to selected regions. The mean and variation of the hapFLK values were estimated using MASS package in R. The hapFLK statistics were then Z-transformed using these parameters and *P*-values were computed assuming a normal distribution in R. False discovery rate (FDR) approach^42^ was then performed using q-value package in R to correct *P*-values for multiple testing. The regions including q-value < 0.05 were considered as potentially selected regions.

#### Gene ontology and functional analysis

We used the biomaRt package of R to find all Ensembl gene IDs overlapped with the candidate regions. Since the domestic dog (*Canis lupus familiaris*) is known as the closest species to mink whose genome has widely been annotated^32^, the gene ontology was conducted based on the reference list of this species. The biological process, molecular function and cellular component terms were assessed for all genes using PANTHER 14.1^43^. Statistical overrepresentation of annotated genes was assessed by Fisher's exact test and corrected by false discovery rate (FDR) procedure. These genes were further investigated by reviewing relevant literatures in relation to the phenotypes or pathways of interest in different groups.

## Results

### Data quality control

**Figure 1:**
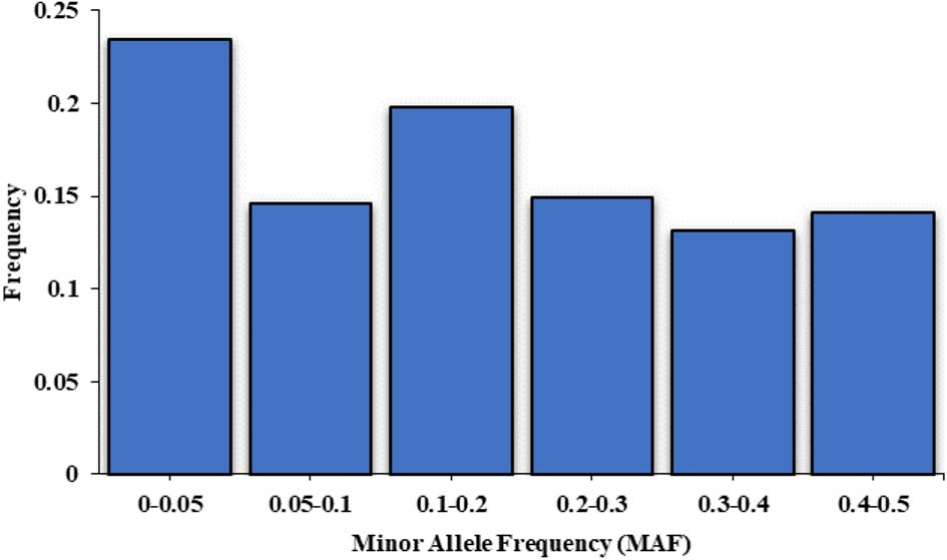
Distribution of minor allele frequencies (MAF) across the data set obtained by GBS in American mink.

The total of 681,936,405 reads was generated with an average of 2,133,590 reads per sample. Overall, 97.12% of reads were mapped to the mink genome assembly with a range of 91.14% to 97.59% among samples. A data set including 62,404 SNPs across 1,834 scaffolds was produced after variant calling. These scaffolds covered 1.75 Gb of the genome, which captured approximately 73% of the 2.4 Gb reference genome. After quality control, 47,800 SNPs from 216 animals were kept for further analyses. The average number of SNPs per scaffold was 34 with the maximum number of 1,320 SNPs on scaffold 10. The average MAF was 0.19 ± 0.15 across all SNPs. Figure 1 presents the proportion of SNPs with different ranges of MAF. A large proportion of SNPs (23%) had MAF < 0.05 whereas the lowest number of SNPs was observed in the range of 0.3 ≤ MAF < 0.4 (13%).

### Genome-wide signatures of selection

#### Genetic differentiation statistics

We measured pooled F_ST_ in 100 kb windows between opposing pairs of each group. The total of 28,305 windows with step sizes of 25 kb was scanned along the genome with the average number of 4.6 ± 7.3 SNPs per 100 kb window. Supplementary Fig. S2 presents the distribution of F_ST_ on the scaffolds showing potential signatures of selection in different groups. Each phenotypic group showed some contiguous windows including high levels of F_ST_, which were potentially the candidate regions of positive selection. A complete list of the top 1% of Z(F_ST_) distribution was provided for each group in the Supplementary Dataset S1. The differentiation of individuals within each group was also assessed based on θ_π_ ratios (i.e. θ_π-Negative_ / θ_π-Positive_). Supplementary Fig. S3–S7 present the overlap of top 1% values between Z(F_ST_) and log_2_ (θ_π ratios_) in different groups. In addition, a complete list of candidate regions along with their positions was provided in Supplementary Table S1. Total number of identified candidate regions was 99 and varied from 12 for antibody titer and viremia-350 groups to 34 for kidney lesion scores (Table 2). These regions harboured 63 genes potentially subjected to selection for response to AMDV infection (Supplementary Table S2). A total of seven candidate regions were shared between at least two phenotypic groups (Table 3). The group with the most shared candidate regions was survival (4 regions). On the other hand, viremia-350 shared only two regions with survival and antibody titer groups.

**Table 2:** Number of candidate regions and genes detected by overlapping Z(F_ST_) and log_2_ (θ_π ratios_) in five differential groups of responses to AMDV-infection.

Group	Number of candidate regions identified	Number of genes
Survival	20	13
Kidney lesions	34	21
Antibody titer	12	8
Virus clearance	21	11
Viremia-350	12	10
Total	99	63

Total do not consider overlap between groups.

**Table 3:** Candidate regions (spans ± 1 Mb) identified by overlapping selective signals of Z(F_ST_) and log_2_ (θ_π ratios_) that were shared by two or more groups.

Scaffolds	Position (bp)	Survival	Kidney lesions	Antibody titer	Virus clearance	Viremia-350
1	23,000,001			×	×	
2	13,750,001	×		×		
2	23,425,001	×		×		×
2	27,325,001	×				×
5	13,850,001	×	×			
6	13,475,001		×		×	
6	18,375,001		×		×	

**Figure 2:**
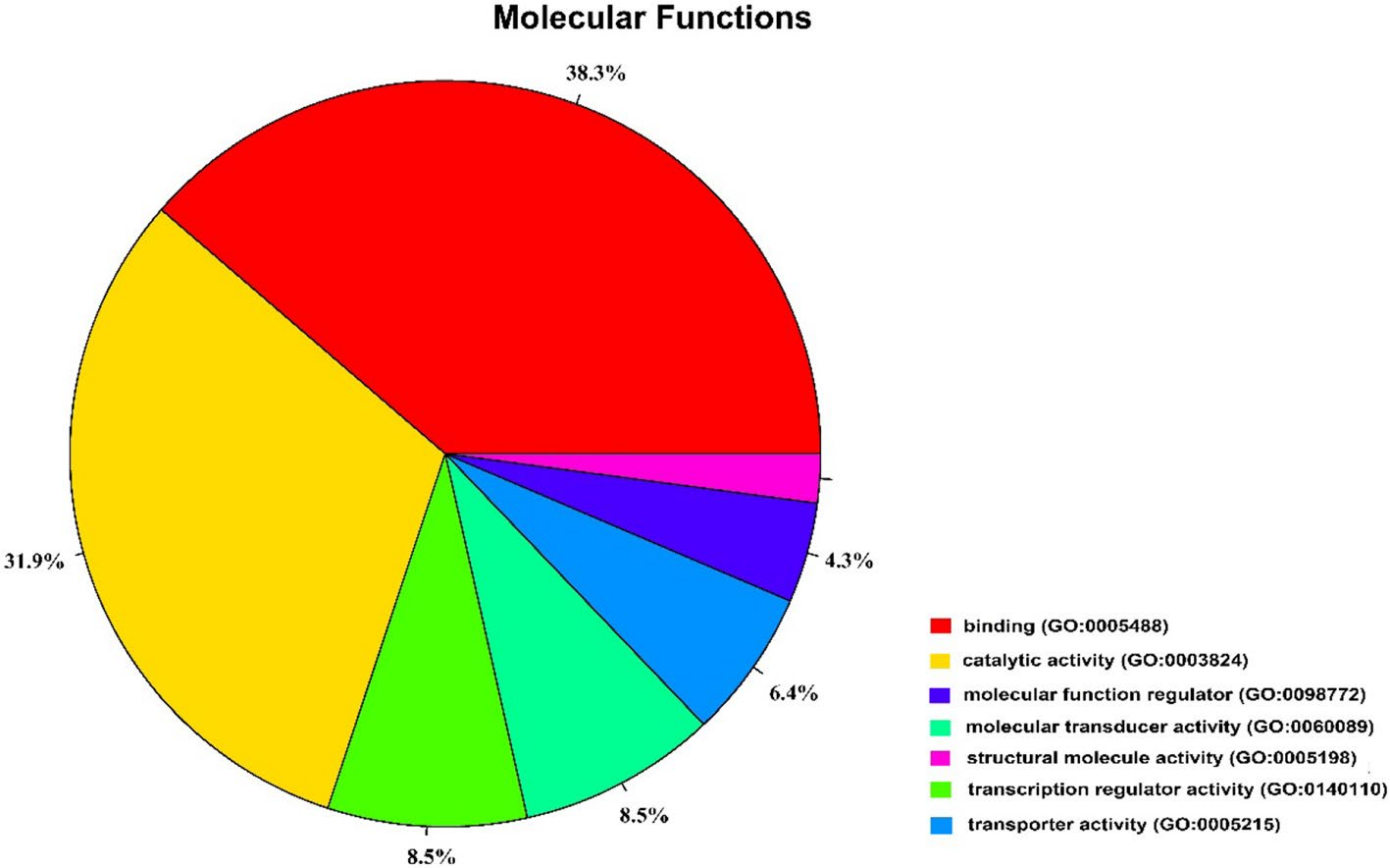
The pie chart of molecular functions attributed to candidate genes detected by overlapping selective signals of Z(F_ST_) and log_2_ (θ_π ratios_).

Figure 2 presents the pie chart of molecular functions attributed to candidate genes in the putative regions of selection. These results indicated that a significant proportion of genes were involved in binding (38.3%) and catalytic (31.9%) activities. In addition, gene ontology analysis resulted in 109 overrepresented (*P* < 0.05) GO enrichment terms related to biological process (Supplementary Dataset S2). For instance, the candidate genes were highly enriched in Wnt signaling, calcium modulating (Wnt/Ca2 +) pathways (GO:0007223), liver regeneration (GO:0097421), animal organ regeneration (GO:0031100), cardiac muscle cell proliferation (GO:0060038), response to isoquinoline alkaloid (GO:0014072), zinc ion transport (GO:0006829), response to progesterone (GO:0032570) and response to stimulus (GO:0050896) , which were relevant to AD-characterized phenotypes (Supplementary Dataset S2). In addition, the gene ontology revealed the biological roles of several genes related to immune system process (*TRAF3IP2*, *WDR7*, *TNFRSF11A*, *SWAP70*, *CBFB*, *IGF2R* and *GPR65*), response to stress (*SULF2*, *CADPS2*, *NOX3*, *GNAO1*, *HSF4*, *AMF*R and *CNOT1*), reproduction (*FBXO5*, *CatSperβ*, *MAS1*, *CATSPER4*, *GOT2* and *IGF2R*) and regulation of nervous system development (*TENM4*, *VSNL1*, *CLSTN1*, *RIT2*, *TCF4*, *SKOR2* and *NDRG4*).

### HapFLK test

**Figure 3:**
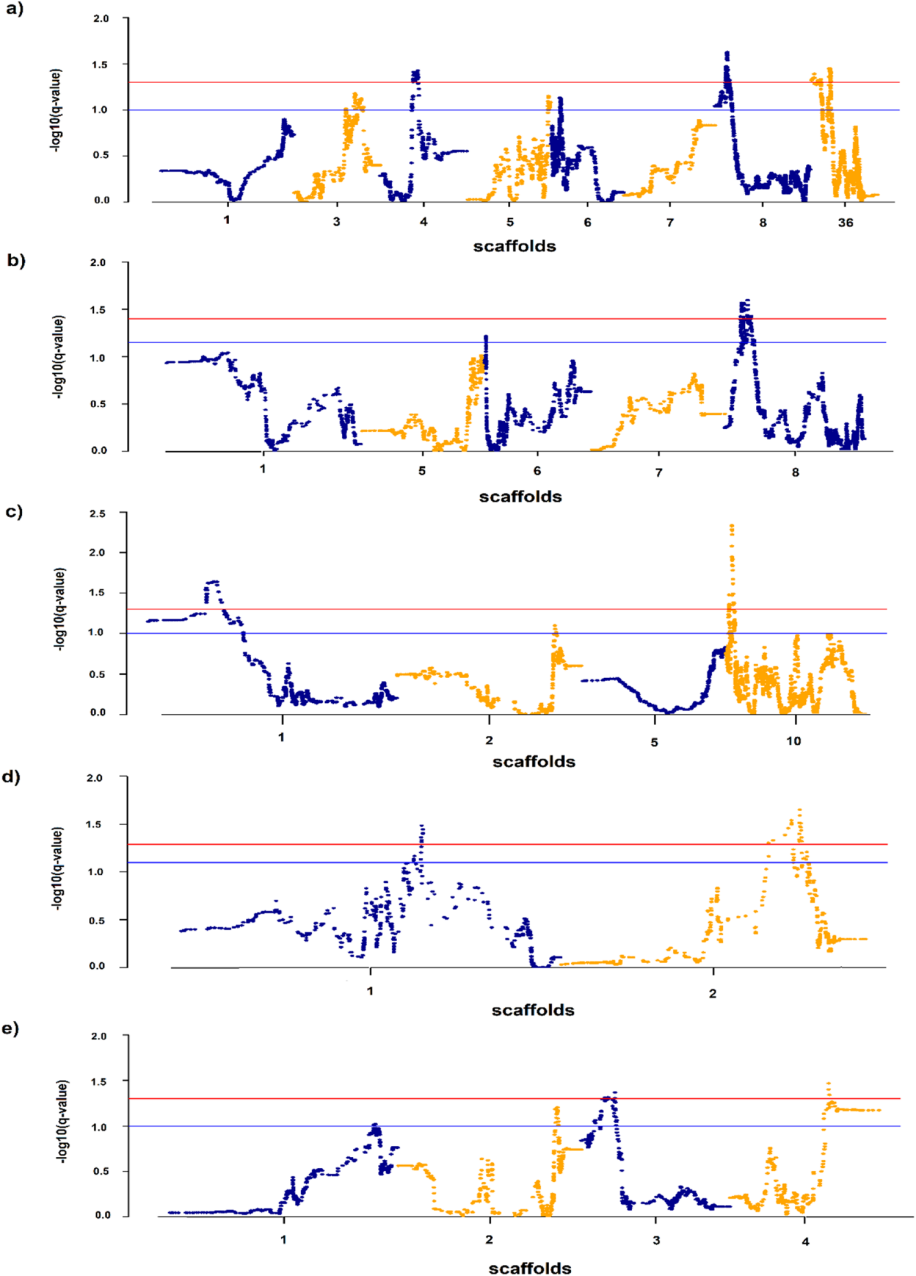
Manhattan plots of hapFLK statistic in different groups: (**a**) kidney lesions (**b**) virus clearance (**c**) survival (**d**) antibody titer and (**e**) viremia-350. Blue and red lines indicate suggestive (q-value < 0.10) and significant (q-value < 0.05) thresholds, respectively.

We used the hapFLK statistic to validate the signatures of selection detected by aforementioned methods. The hapFLK values were computed only for scaffolds containing significant candidate regions from previous approach. Figure 3 presents − log_10_ (*q*-value) of hapFLK statistics per each genomic position in different groups. Table 4 presents the putative regions identified based on hapFLK test along with the candidate genes located in these regions. Among all ten candidate regions detected by hapFLK test, five regions were overlapped with those positions identified by previous approaches (Table 4). The GO enrichment analysis indicated that selected regions were involved in 71 biological process GO terms (Supplementary Dataset S2). A fold enrichment > 100 was observed for some biological processes such as neutrophil degranulation (GO:0043312), liver regeneration (GO:0097421), neutrophil activation involved in immune response (GO:0002283), leukocyte degranulation (GO:0043299), androgen receptor signaling pathway (GO:0030521) and macrophage differentiation (GO:0030225).

**Table 4:** Candidate regions identified by hapFLK test along with genes involving in these regions.

Scaffolds	Position (bp)	Group	Lowest q-value	Gene symbols	Overlapping status^a^
1	9,129,632–9,979,233	Survival	0.023	*BMP4*, *CDKN3*, *CNIH1*, *GMFB*, *CGRRF1*, *SAMD4A*	–
10	915,739–1,126,382	Survival	0.004	*AJAP1*	–
1	23,046,774–23,187,210	Antibody titer	0.034	*SRSF5*, *SLC10A1*	×
2	23,424,424–23,969,935	Antibody titer	0.022	*RNF165*, *LOXHD1*, *SKOR2*	×
3	4,297,807–4,310,786	Viremia-350	0.048	*WWP2*	–
4	16,076,915–16,196,438	Viremia-350	0.034	*PTPRZ1*	–
4	10,514,110–10,841,037	Kidney lesions	0.037	*KLHDC10*, *ZC3HC1*, *UBE2H*	×
8	3,478,058–4,850,122	Kidney lesions	0.023	*SULF2*, *EYA2*, *SLC2A10*, *TP53RK*, *SLC35C2*	×
36	5,250,514–5,725,445	Kidney lesions	0.035	*ATE1*, *NSMCE4A*, *TACC2*	–
8	3,620,504–5,308,341	Virus clearance	0.025	*SULF2*, *EYA2*, *SLC2A10, TP53RK*, *SLC35C2*, *PLTP*, *MMP-9*, *CTSA*, *ELMO2* , *OCSTAMP*, *UBE2C*, *ACOT8*	×

^a^Overlaps with those regions detected by integrated measurement of F_ST_ and θ_π_.

## Discussion

The SNP data set generated by GBS technique was used to scan the American mink genome for positions that might have been targeted by selection for response to AMDV infection. A large proportion of sequencing reads (on average, 97.12%) were mapped to the mink genome reference, which revealed the efficiency of methodology applied in this study. The GBS approach was previously used to identify markers associated with body size and pelt length in American mink^44^. The number of detected SNP markers in the current study (62,404) is higher than that (34,816) reported by Cai et al.^44^, that might mainly be attributed to different restriction enzymes applied to digest DNA sequences in these studies. Although, similar to the results by Cai et al.^44^, high percentage (23%) of SNPs had MAF < 0.05, the uniformity of MAF distribution was higher in the current study (Fig. 1). In addition, the GBS method has previously been used to identify the signatures of selection in other domestic animals e.g. Duroc pigs^45^ and Dromedary camels^46^. Our results suggested that GBS technique is an efficient approach to perform genome-wide studies in American mink, where there is no SNP panel available so far.

In the current study, the phenotypic variation within the population was used to classify the animals to different subpopulations. Use of inter-population selection signatures approach can be exemplified by the studies of litter size trait in dairy goat^47^, backfat thickness in Yorkshire pigs^48^, adaptation to different environments in Atlantic salmon^49^ and disease risk in Japanese people^50^. The current study revealed several loci which have likely been under selection by combining F_ST_ and θπ scores, and validated by haplotype-based (hap-FLK) tests. Given the fact that it was difficult to determine a strict threshold to distinguish homozygous regions undergoing selection from those caused by genetic drift, we combined the top 1% genomic regions obtained by both F_ST_ and θπ to narrow our results. In addition, since the unknown demography of the population made it difficult to interpret the distribution of statistics under the null hypothesis of no selection and weakened the detection of signatures of selection^51^, we integrated the statistical approaches to increase the likelihood of pinpointing to genomic regions that most likely contributed to true signatures of selection within the population.

Despite the existence of a large number of non-viremic samples at day 350 pi (156 individuals), most of these animals (128 individuals) harboured the virus (PCR positive spleen) when euthanized (Table 1). It is well established that AMDV replication peaks around day 10 pi and then declines^1^, which causes irregular and short-lived viremia in most mink^13,26,27,52,53^. The virus remains in the lymphoid organs, such as the spleen, which are the primary sites of virus replication and sequestration^1^, and can be detected in the lymphoid organs long after the drop in viral replication and the absence of viremia^26^. Only a small number of mink are able to clear the virus, as shown in the current and previous studies^13,15,54^.

A total of 99 candidate selection regions were identified on ten scaffolds by overlapping the top 1% of Z(F_ST_) and log_2_ (θπ ratios). These regions were annotated by 63 genes based on the Ensembl genome database. Gene ontology indicated that most of these genes (38.3%) were enriched in binding activity (GO:0,005,488), which can be attributed to the mechanisms involved in the disease progress. The AMDV infection causes an immune complex-mediated disease, which is characterized by hypergammaglobulinemia and leads to penetrate viral particles into target cells through virus-antibody complexes^55,56^. Therefore, reactions against developing AD might be associated with genes controlling binding activity. Furthermore, high enrichment of genes involved in catalytic activities might be related to wide histopathological reactions of liver and kidneys due to AD progression^57^. Furthermore, several biological process GO terms were found in connection with AD target phenotypes (Supplementary Dataset S2). For example, it was demonstrated that Wnt/Ca2 + signalling pathway plays a major role in inflammatory responses and co-operative activation of innate and adaptive immunity^58^.

### Immunity responses

Since AD causes a virus-induced disorder of the immune system, we focused on genomic regions containing genes related to immune responses. Our results identified seven key genes (*TRAF3IP2*, *WDR7*, *SWAP70*, *TNFRSF11A*, *CBFB*, *IGF2R*, and *GPR65)* related to immune system process, which might play causal roles in immune-mediated responses to AMDV infection. The *TRAF3IP2* gene was detected at scaffold5: 9.51–9.55 Mb by integrated analysis of F_ST_ and θπ in kidney lesions group. The *TRAF3IP2* gene encodes nuclear factor-kappa-B (*NF-κB*) activator 1 (*Act1*), known as the protein that is found in a range of immune cells such as epithelial cells, B cells, T helper (Th) cells, and neutrophils cells^59,60^. It has been suggested that the *TRAF3IP2* gene played a significant role in the homeostasis of B cells^61^ and acted as a positive regulator in the *IL17*–dependent signaling pathway associated with autoimmunity and inflammatory diseases^62^. Similar to *TRAF3IP2*, the *SWAP70* (scaffold36: 16.85–16.92 Mb, kidney lesions group) is also a coding gene contributing in *NF-κB* signaling pathway. This gene restricts spontaneous maturation of dendritic cells and is associated with the capacity to induce immune responses^63^. The role of *SWAP70* was also reported in c-kit receptor signaling, which was the key pathway in proliferation and differentiation of mast cells^64^. The *TNFRSF11A* gene is located on scaffold6: 13.93–13.96 Mb (kidney lesion group), and involves in autoinflammatory disorders and dysfunction of the innate immune system^65^. The *WDR7* gene was identified on scaffold6: 18.16–18.52 Mb and shared between the results obtained in both virus clearance and kidney lesions groups, which are the two important measures associated with tolerance to AD. It was reported that *WDR7* plays a regulatory role in the endoplasmic reticulum contributing to protein processing and secretion^66^. The efficiencies of the endoplasmic reticulum can influence immunity system e.g. the maturation process of B-cells to immunoglobulin secreting plasma cells^67^. The *CBFB* was the other gene related to immune responses, which was detected on scaffold3: 6.72–6.78 Mb in viremia-350 group. The *CBFB* gene involves in the pathway of making a protein called core binding factor subunit beta (*CBFB*), which is known to contribute in autoimmunity and inflammation^68,69^. In humans, it was revealed that *CBFB* is required for expression of the *HIV-1*-restrictive *APOBEC3* gene repertoire^70,71^. The *IGF2R* gene was identified on scaffold5: 22.64–22.74 Mb in virus clearance group and it was shown to enhance the regulatory T-cell functions^72^ and functions of antigen-specific regulatory B cells^73^. Finally, a region containing the *GPR65* gene was detected on scaffold1: 27.71–27.72 Mb in survival group, which was related with the immune reactions to AMDV infection. The *GPR65* gene plays a central role as the critical regulator of pro-inflammatory T cell (*Th17*) pathogenicity^74^. This key gene also involves in immune response by maintaining lysosome function and may have a function in activation-induced cell death or differentiation of T-cells^75,76^.

### Reproductive performances

It has been confirmed that AMDV infection causes reduced litter sizes and pregnancy rates, and increases abortion and mortality rates in American mink^5^. Our results revealed multiple genes associated with reproductive functions, which might play potential roles connected to these impacts. For instance, it was shown that *FBXO5* (scaffold5: 16.61–16.62 Mb, virus clearance group) is strongly expressed in oocyte and blastocyst tisseus^77,78^ and is a down-regulated gene arresting the differentiation and growth of the human cumulus cell in the periovulatory period^79^. Furthermore, the *CatSperβ* gene was detected in virus clearance group on scaffold1: 24.56–24.67 Mb and was associated with sperm cell hyperactivation. It was shown that this gene is essential for sperm motility as well as the preparation of sperm for fertilization^80^. Similarly, the *CATSPER4* gene (scaffold10: 17.41–17.42 Mb, survival group) is related with the male infertility^81^. The *IGF2R* gene was identified on scaffold5: 22.64–22.74 Mb in virus clearance group and it seems that the expression of this gene plays critical roles in pre-implantation of embryos^82^ and birth weight^83^. The effect of this gene was also reported for growth traits in Egyptian buffalo^84^. Finally, it was revealed that the *GOT2* gene (scaffold3: 13.84–13.87 Mb, kidney lesions group) is expressed in placenta and can be influenced by maternal factors^85^. These results revealed the possible connection between potentially selected genes for response to AMDV infection and reproductive performances in American mink.

### Other responses

Our results revealed several candidate genes related to heart (*FHOD3*, *TENM4*, *WNT11* and *NDRG4*), liver (*SULF2*, *SRSF5* and *IGF2R*) and kidney (*SULF2* and *WNT11*) developments (Supplementary Dataset S2), which might be associated with the clinical signs caused by AD in these tissues^86^. Moreover, many candidate genes were related to response to stress (*SULF2*, *CADPS2*, *NOX3*, *GNAO1*, *HSF4*, *AMF*R, and *CNOT1*) and regulation of nervous system (*TENM4*, *VSNL1*, *CLSTN1*, *RIT2*, *TCF4*, *SKOR2*, and *NDRG4*), which might indicate the significant role of response to stimulus during the AD development (Supplementary Dataset S2).

### Candidate regions shared between various phenotypic groups

In total, seven overlapped regions were observed between at least two groups of individuals, providing independent supports for the candidate regions (Table 3). The most shared selected regions were identified in the survival group (4 regions), suggesting that this group can be used as the best measure to scan the genomic signatures of selection in American mink for response to AD. On the other hand, the lowest number of shared selected regions was revealed by viremia-350 group.

We identified ten candidate genes on the seven putatively selected regions shared between at least two groups. Four genes (*SLC39A9*, *PLEKHD1*, *SRSF5* and *SLC10A1)* were shared among the putative positions detected on scaffold1 by antibody titer and virus clearance groups (Table 3). The *SLC39A9* gene (scaffold1: 22.80–22.86 Mb) encodes the zinc transporter *ZIP9* protein, which regulates zinc homeostasis in the secretory pathway^87^. The *SLC39A9* gene played an essential role in regulating the activations of Akt and Erk in B-Cell receptor signaling pathway in chicken DT40 cells^88^. This gene was also identified as a candidate gene involving in the molecular mechanisms underlying chikungunya virus infection in human^89^. The *PLEKHD1* gene (scaffold1: 22.87–22.90 Mb) was located on the upstream of *SLC39A9* gene and potentially interacted with *SLC39A9* by forming chimeric genes^90^. The *SRSF5* gene (scaffold1: 23.11–23.12 Mb) constitutes a protein which is a member of the serine/arginine (SR)-rich family of pre-mRNA splicing factors^91^. The *SRSF5* is known as a main regulator of human immunodeficiency virus type 1 (HIV-1) mRNA splicing^92^ and promotes the translation of un-spliced HIV-1 RNA^93^. In addition, it was identified as insulin-induced protein in regenerating liver^94^. The *SLC10A1* gene was also detected on the same region (scaffold1: 23.12–23.15 Mb) and predominantly expressed on hepatic basolateral membranes^95^. This gene plays key roles as bile acid transporter^96^ as well as the receptor for hepatitis B and D virus^97^. It was suggested that product of this gene regulated the innate antiviral responses in liver^98^.

Two genes (*GALNT1* and *SLC39A6)* were found within the putatively selected regions on scaffold2: 13.75–14.40 Mb, which were detected by both survival and antibody titer groups (Table 3). It was suggested that expression of *GALNT1* gene was required for the normal heart valve development and cardiac function^99^. The *SLC39A6* gene encodes zinc transporter ZIP6, which is essential for the function of the lymphocyte activation machinery^100^. Moreover, the *SLC39A6* plays a functional role in preparation of monoclonal antibodies^101^, which could modulate anti-AMDV antibody production. Another candidate region located on scaffold 2 (23.42–24.15 Mb) was validated by the analyses of survival, viremia-350 and antibody titer groups (Table 3). This selected region contains *RNF165*, *LOXHD1* and *SKOR2* genes. The *RNF165* and *SKOR2* genes were annotated to be involved in response to stimulus. The *RNF165* gene involves in epigenetic programming of T-cell phenotypes during early development in humans^102^. The *SKOR2* gene is a member of the SKI protein family, which was known as one of the major negative regulators of the transforming growth factor-β (TGF-β) signaling pathway. It was indicated that loss of TGF-β signaling was associated with inflammation and autoimmune diseases^103^, which is in accordance with the fact that AD is a virus-induced disorder of the immune system and autoimmune disease^1^. Moreover, *SKOR2* gene was associated with female infertility in inbred mice^104^. The *LOXHD1* gene encodes a highly conserved protein that localizes along the plasma membrane of stereocilia in the hair cells. This gene was expressed in the mechanosensory hair cells and was related to auditory defects in mice, showing that it is required for normal hair cell function in the inner ear^105^. In addition, mutations within *LOXHD1* were related to nonsyndromic hearing loss in humans^106^.

Finally, two 13.47–13.95 Mb and 18.37–19.42 Mb regions on scaffold 6 were shared between the suggestive positions detected by kidney lesions and virus clearance groups. The presence of common genes controlling these two phenotypes is reasonable since kidney lesions are caused by deposition of antibody-virus complexes^29,107^, which disappear when animals clear the virus. The region of 13.44–13.65 Mb contains the *PHLPP1* gene, which regulates the transcription of genes involved in magnitude and duration of inflammatory signaling and innate immune responses^108^. It was suggested that reduced expression of *PHLPP1* enhanced the antiapoptotic B-cell receptor signal in chronic lymphocytic leukemia B-cells^109^. The other suggestive selected region was detected on scaffold6: 18.37–19.42 Mb, shared between kidney lesions and virus clearance groups. This region contains the *WDR7* gene that plays a role in immunity system as described in the previous section. In addition, we found the *TCF4* gene located in the downstream of this region (scaffold6: 19.40–19.79 Mb), which was known as an immunoglobulin enhancer expressing predominantly in pre-B-cells^110^. The protein encoded by this gene (transcription factor E2-2) was identified as the critical regulator of plasmacytoid dendritic cells (PDCs) development. The PDCs provide a unique immune cell type specialized in type I interferon (IFN) secretion in response to viral nucleic acids^111^.

### Validation by HapFLK test

Given the fact that the number of small scaffolds was high in the data set, the hapFLK tests were simply restricted to those regions detected by differentiation measures in the previous step. As expected, a larger number of candidate regions were detected by integrated measurement of differential statistics (F_ST_ and θπ) compared to those identified by haplotype-based hapFLK test. In fact, hapFLK test can only identify selected regions with long haplotypes whereas differential methods are more sensitive to detect shorter candidate regions^112^. However, 50% of suggestive regions detected by hapFLK approach were also found by integrated measurement of F_ST_ and θπ, indicating that these positions can be considered as more reliable candidates of selective sweeps for AD in American mink. The haplotype region of scaffold8 (3.47–5.30 Mb) was detected by hapFLK tests of both kidney lesions and virus clearance groups, which was also validated by the genetic differential analyses (Table 4). Our results indicated that this region contained multiple key genes related to immune system process including *MMP-9*, *CTSA*, *ELMO2*, *OCSTAMP* and *ACOT8*. Interestingly, it has been shown that the *MMP-9* gene plays a critical role in the clearance of autoantigens, autoantibodies and immune complexes in plasma. There is evidence that the lack of *MMP-9* could lead to increase in the levels of immune complexes in plasma and local complement activation in spleen and kidneys^113,114^, suggesting that this gene can be a prominent candidate for variation in virus clearance among mink. This gene was also known to be involved in the ovarian responses to gonadotropins, sex hormones, and *TGFB1* in Chicken^115^. The *CTSA* gene is expressed in primary antigen-presenting cells^116^ and the *ELMO2* gene is essential in phagocytosis and cell migration^117^. It was revealed that the *OCSTAMP* gene suppresses M1 pro-inflammatory state by inducing a phenotypic switch in macrophage polarization^118^. Finally, the *ACOT8* gene encodes a protein binding to the HIV-1 protein Nef and participates in Nef-mediated MHC-I downregulation and the prevention of T-cell activation^119^.

Another candidate region supported by all statistical approaches was detected on scaffold4: 10.51–10.84 Mb. Two genes, *KLHDC10* and *UBE2H*, were identified in this selected region, which were associated with immune responses. It was suggested that *KLHDC10* deficiency protected mice against TNFα-induced systemic inflammation^120^. The *UBE2H* gene encodes ubiquitin-conjugating enzyme E2 H. The ubiquitin plays a crucial role in cellular mechanisms involving in proteins modifications to target abnormal or short-lived proteins for degradation^121^. In addition, deregulated ubiquitination events were related to autoimmune and inflammatory responses^122^.

Finally, two regions including scaffold1: 23.04–23.18 Mb and scaffold2: 23.42–23.96 Mb were detected by hapFLK test of antibody titer group, which were also supported by the integrated analysis of F_ST_ and θπ in two or more of the phenotypic groups (Table 4). These results suggested that these regions were strong candidates of selection for AD tolerant mink. Five key genes, *RNF165*, *LOXHD1*, *SRSF5*, *SLC10A1* and *SKOR2,* were shared among these suggestive regions. These genes, as described in the previous sections, might involve in critical responses to AMDV infection, such as immune responses, virus–host interactions, reproductive process, liver regeneration and response to stimulus.

Although there is no study on detecting genomic signatures of selection in American mink, this approach has been used to analyze resistance against diseases in other domestic animals e.g. susceptibility to gastrointestinal nematodes in sheep^123^ and resistance to Marek’s disease in inbred chicken lines^124^. In addition, recent studies indicated that pathogen-driven selection has played a strong role in human genome evolution^125^. For instance, strong selection signatures were identified in multiple loci in relation with human immunology^126^, adaptation to pathogen pressure^127^, innate immunity^128^ and histocompatibility complex (MHC) regions^129^. We identified numerous well-documented genes related to immune responses, reproduction, liver and kidney functions which might contribute to AD tolerance in American mink. The combination of several groups of AD-related phenotypes as well as integration of different statistical approaches were used to support the results of this study. However, owing to the lack of complete/correct genome annotation in American mink, the issue of false positive and gene missingness could not be assessed in this study. Thus, further investigations would be required to validate the role of these putatively selected regions in AD control. The collection of suggested genes in this study presents a foundation for development of molecular approaches to unveil the response of mink to AMDV infection. In addition, given the fact that the lack of chromosome-scale information restricted the analyses of this study to larger scaffolds (> 10 Mb), the availability of chromosome-scale genome assembly can improve the detection of selection signatures in future studies.

The AMDV is known as a parvovirus with a high degree of variability that can infect a wide range of the *Mustelidae* family e.g. European mink, ferrets, Eurasian otters, stone martens, polecats and pine martens. Moreover, the occurrence of AMDV infection was reported in other small carnivores e.g. striped skunks, foxes, common genets and raccoons^28,130^. Accordingly, both host factors and viral strains can affect the pathogenesis of AMDV in the infected animals. Obviously, the results of this study could be extended for further research in the other susceptible species. It is suggested to take advantage of transcriptome analysis in the other related species to investigate the candidate genes revealed in the current study.

Selection for tolerant mink is certainly a feasible approach to control the AMDV infection on mink farms. Genomic selection can be applied as a useful breeding strategy to improve the economically important traits in the mink industry^131^. Detection of numerous loci in this study implies that genomic selection would be an effective approach to increase the tolerance against AMDV infection in mink. Although results of the current study confirmed that survival phenotype could provide the most effective measurement to reveal the selected regions of genome for response to AMDV infection, collecting such records on farms cannot be practical owing to high costs and technical issues. The antibody titer test is not only a practical measurement in farms, but also contributed to identify significant candidate regions in the current study, making it a suggestive phenotype to select tolerant animals in farms.

## Conclusion

This study provided the first map of underlying selection signals of AD in American mink. The integrated results of genetic differentiation analyses as well as haplotype-based tests revealed numerous well-known genes related to AD phenotypes. Two strongly selected regions were detected on scaffold1: 23.04–23.18 Mb and scaffold2: 23.42–23.96 Mb, which contained the critical genes involving in immune responses, virus–host interactions, reproductive process, liver regeneration and response to stimulus. The functional annotation supported the association of AD phenotypes with genomic regions detected in the current study. However, further investigation will be essential to validate the functions of these putatively selected regions for response to AMDV infection. Detection of multiple loci undergoing the selection for AMDV infection indicated that genomic selection can be applied as a feasible approach to control this disease. The survival phenotype was recognized as the best measure to reveal genomic signatures of selection for response to AMDV infection. However, the antibody titer test could be suggested as the applicable measurement to develop genomic selection for increasing AD tolerance in mink farms.

## Supplementary Information

Supplementary Information 1.

Supplementary Information 2.

Supplementary Information 3.

## Data Availability

The datasets generated/analyzed for this study are available from the FigShare Repository: https://doi.org/10.6084/m9.figshare.9757784.
