# Supplementary material for: Detection of selection signatures for response to Aleutian mink disease virus infection in American mink
Source: Sci Rep. 2021 Feb 3;11:2944. doi: 10.1038/s41598-021-82522-8 (PMC7859209; doi:10.1038/s41598-021-82522-8)
Supplement: Supplementary file 3 — Supplementary Information 3. [file 41598_2021_82522_MOESM3_ESM.pdf]

Detection of selection signatures for response to Aleutian mink disease virus infection in American mink

Karim Karimi[1], A. Hossain Farid[1], Sean Myles[2] & Younes Miar[1*]

[1]Department of Animal Science and Aquaculture, Dalhousie University, Truro, NS, Canada; [2]Department of Plant, Food, and Environmental Sciences, Dalhousie University, Truro, NS, Canada

[*]Corresponding author:
Younes Miar

miar@dal.ca

This file includes: Supplementary Figure S1-S7 and Supplementary Table S1-S2.

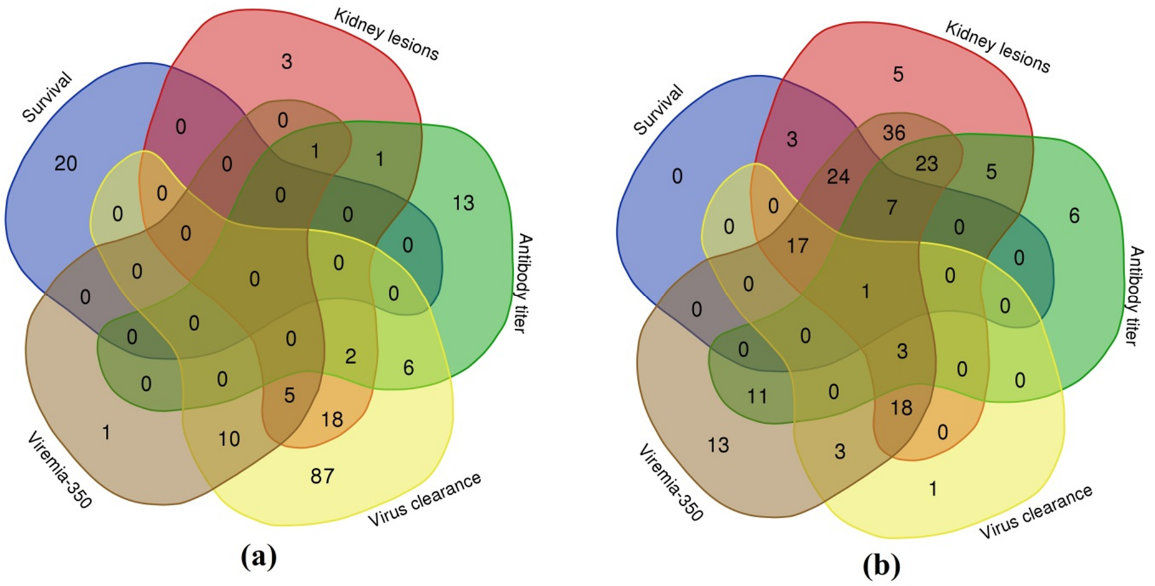

Supplementary Figure S1. The Venn diagrams of the number of individuals shared among five groups of the responses to AMDV infection. The positive (**a**) and negative (**b**) subgroups were presented separately.

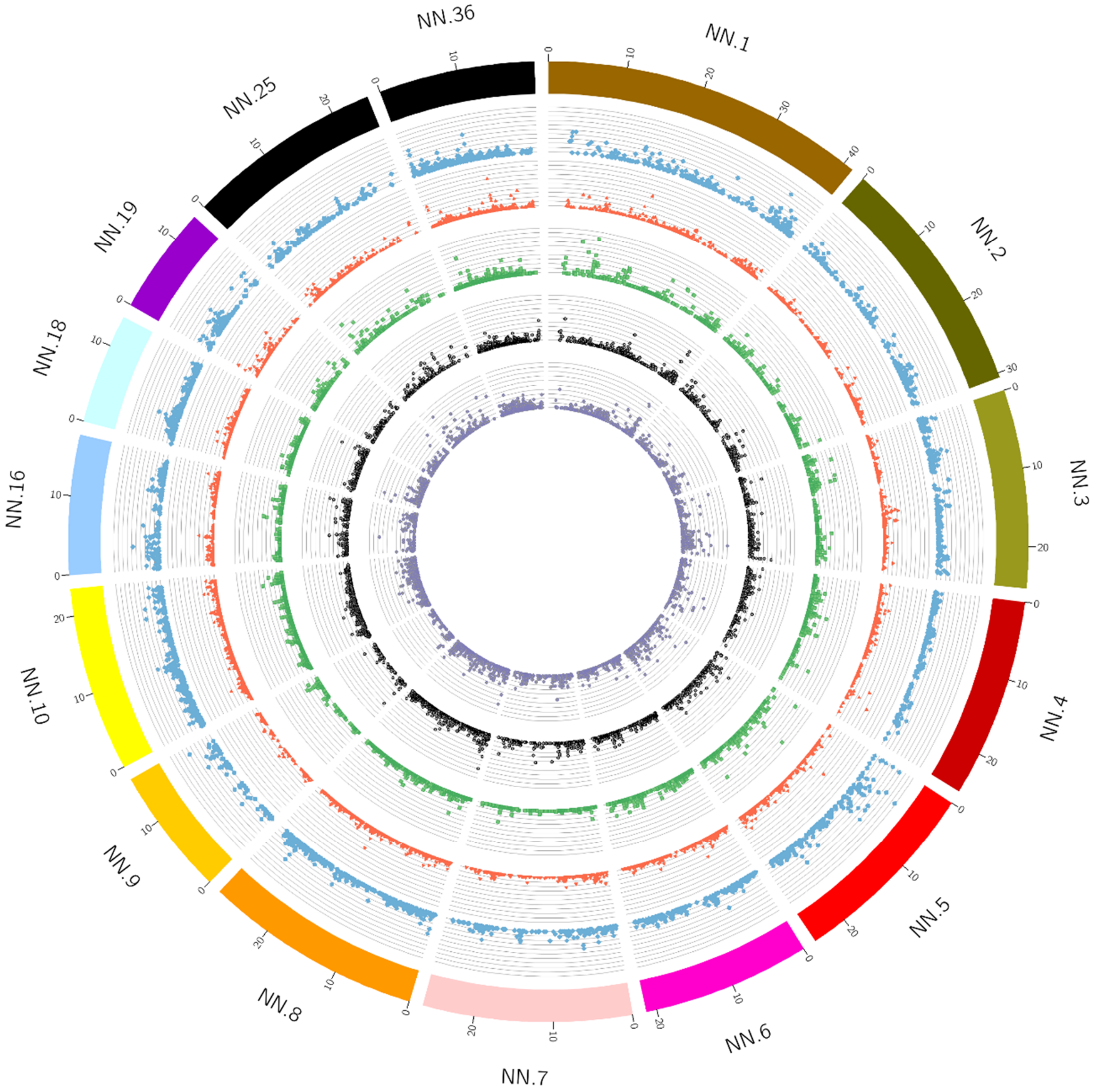

Supplementary Figure S2. Circos plot presenting genome-wide distribution of F_{ST} across the candidate scaffolds in different groups: antibody titer (blue layer), virus clearance (red layer), survival (green layer), kidney lesions (black layer) and viremia-350 (purple layer). The 'NN' shows "*Neovison Vison* scaffold" for easy display on figure.

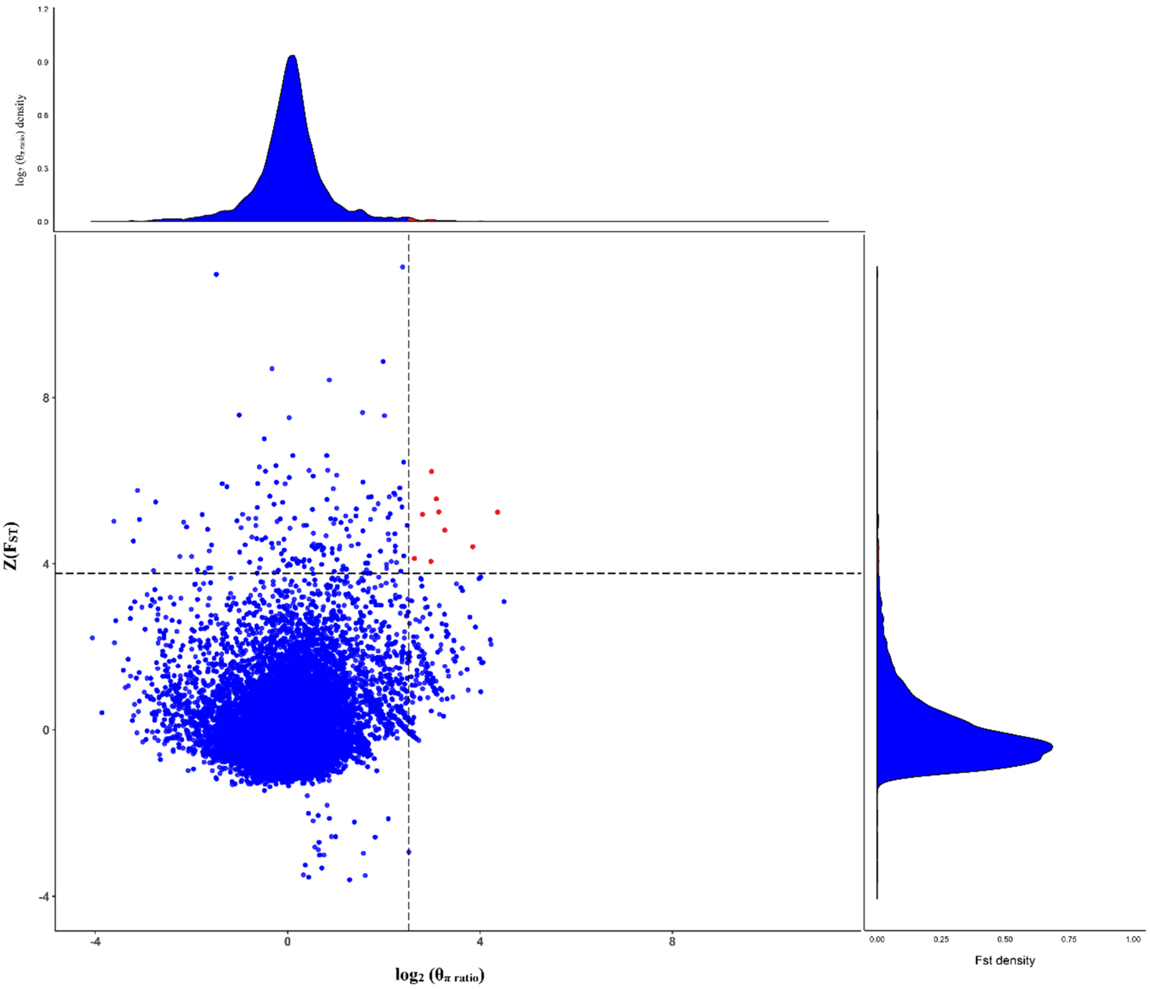

Supplementary Figure S3. Distribution of θπ ratios (θπ $_{\text{Negative}}$ / θπ $_{\text{Positive}}$) and Z(F_{ST}) values in survival group. Red points correspond to the overlap of top 1% of empirical *log*$_2$ (θπ ratios) and Z(F_{ST}) distributions. The two horizontal and vertical dashed lines represent the top 1% value of Z(F_{ST}) (3.77) and *log*$_2$ (θπ ratios) (2.52), respectively. Only scaffolds >10 Mb were used for analyses.

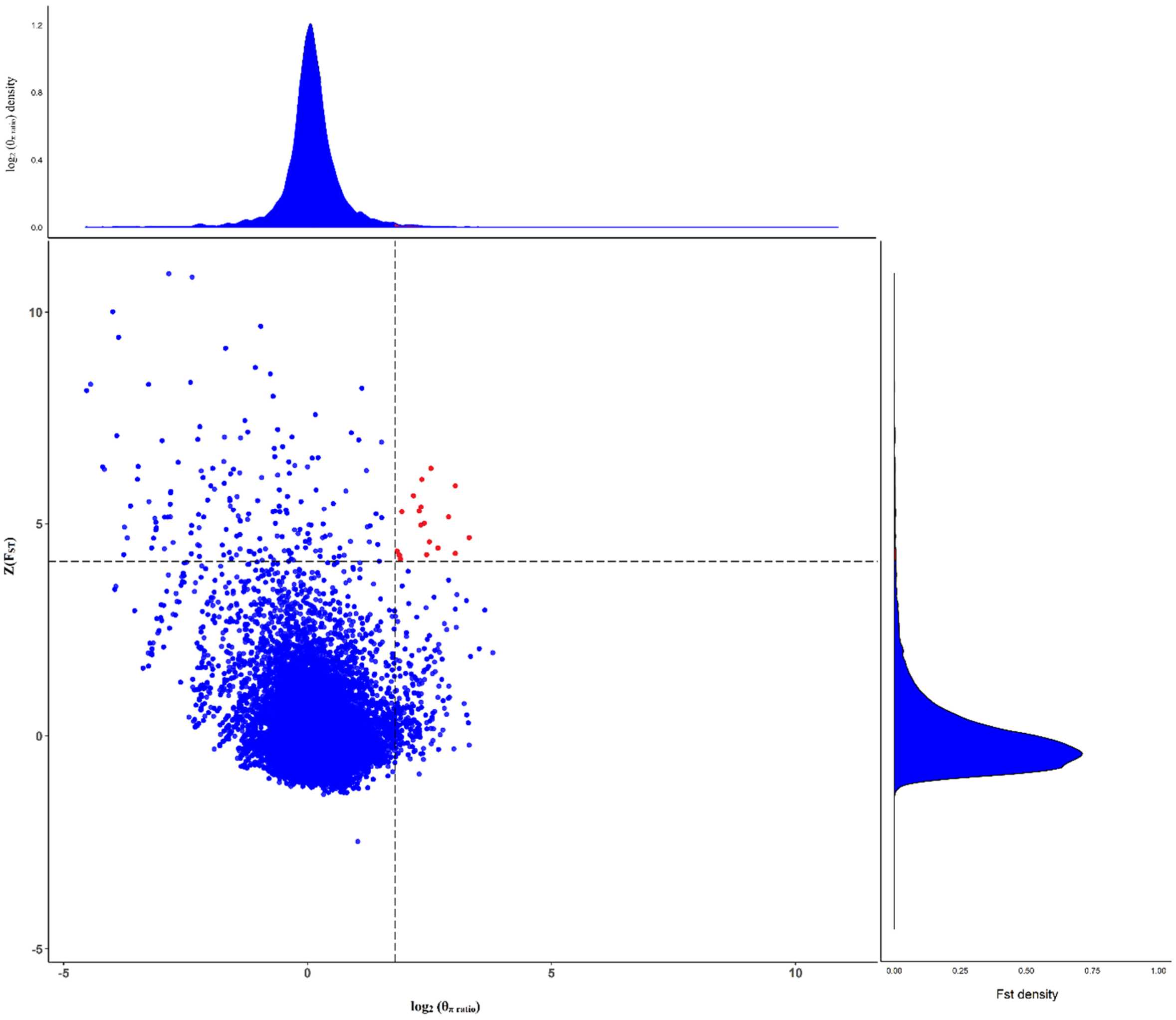

Supplementary Figure S4. Distribution of θπ ratios (θπ $_{\text{Negative}}$ / θπ $_{\text{Positive}}$) and $Z(F_{ST})$ values in kidney lesions group. Red points correspond to the overlap of top 1% of empirical log_2 (θπ ratios) and $Z(F_{ST})$ distributions. The two horizontal and vertical dashed lines represent the top 1% value of $Z(F_{ST})$ (4.11) and log_2 (θπ ratios) (1.79), respectively. Only scaffolds >10 Mb were used for analyses.

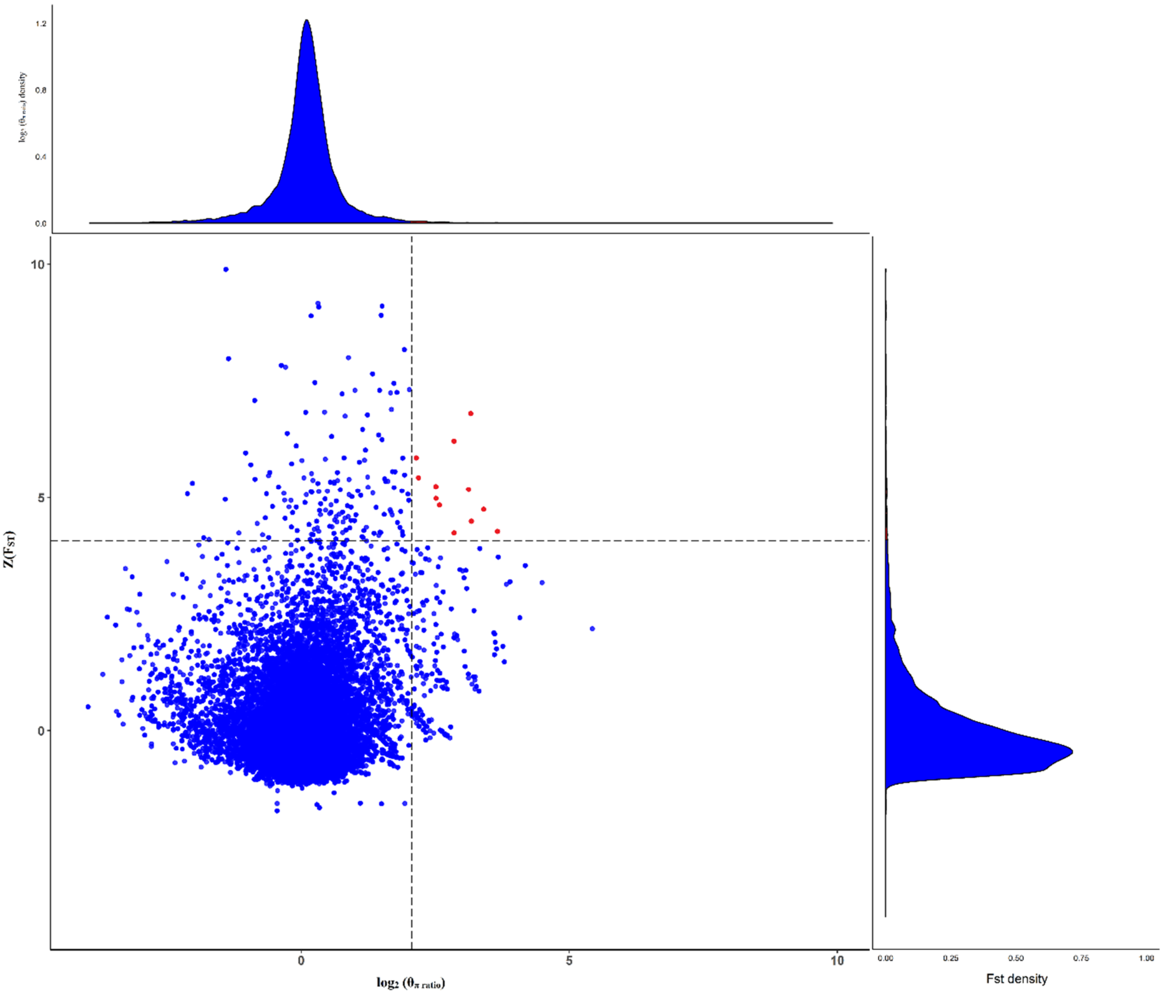

Supplementary Figure S5. Distribution of θπ ratios (θπ $_{Negative}$ / θπ $_{Positive}$) and Z(F_{ST}) values in virus clearance group. Red points correspond to the overlap of top 1% of empirical *log*$_2$ (θπ ratios) and Z(F_{ST}) distributions. The two horizontal and vertical dashed lines represent the top 1% value of Z(F_{ST}) (4.07) and *log*$_2$ (θπ ratios) (2.06), respectively. Only scaffolds >10 Mb were used for analyses.

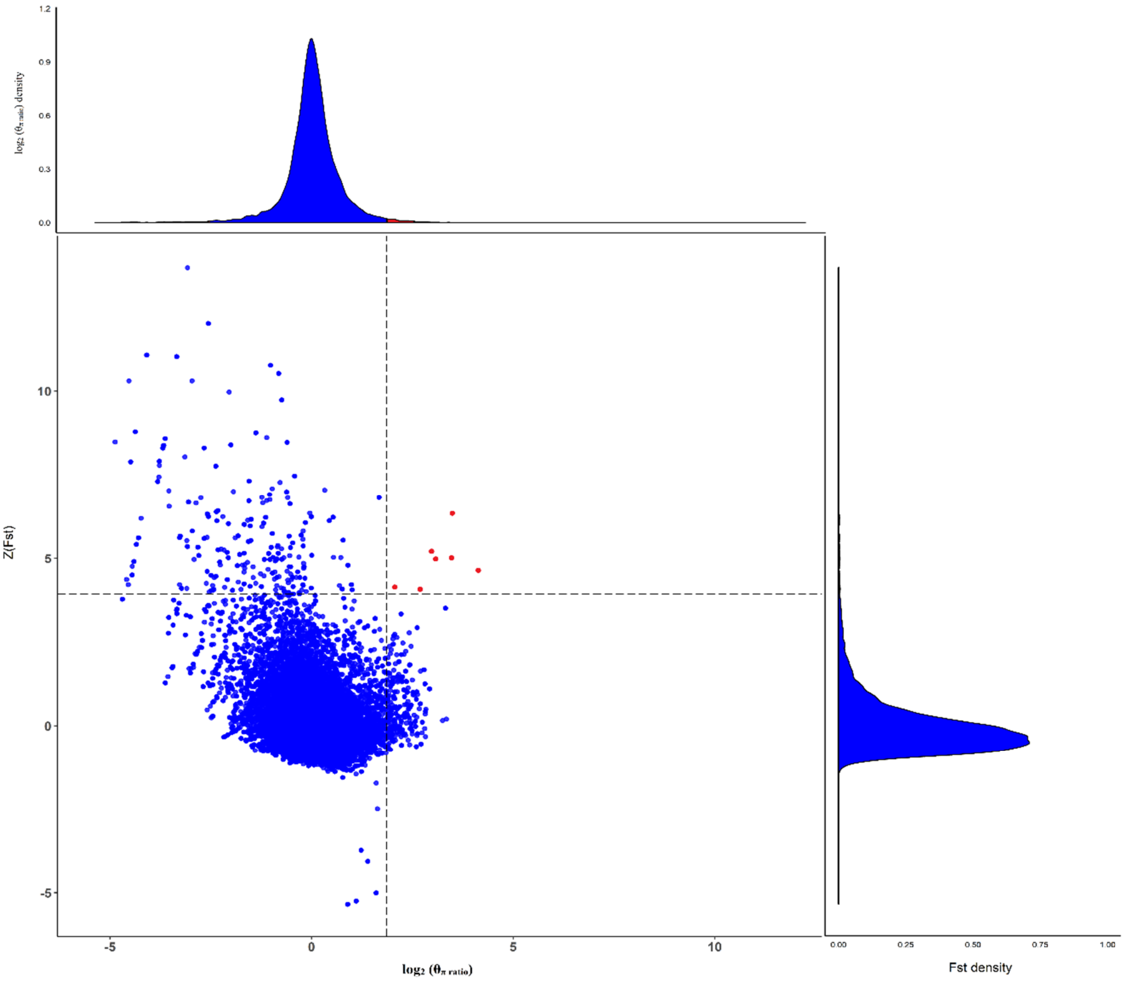

Supplementary Figure S6. Distribution of θπ ratios (θπ $_{\text{Negative}}$ / θπ $_{\text{Positive}}$) and Z(F_{ST}) values in viremia-350 group. Red points correspond to the overlap of top 1% of empirical log_2 (θπ ratios) and Z(F_{ST}) distributions. The two horizontal and vertical dashed lines represent the top 1% value of Z(F_{ST}) (3.93) and log_2 (θπ ratios) (1.86), respectively. Only scaffolds >10 Mb were used for analyses.

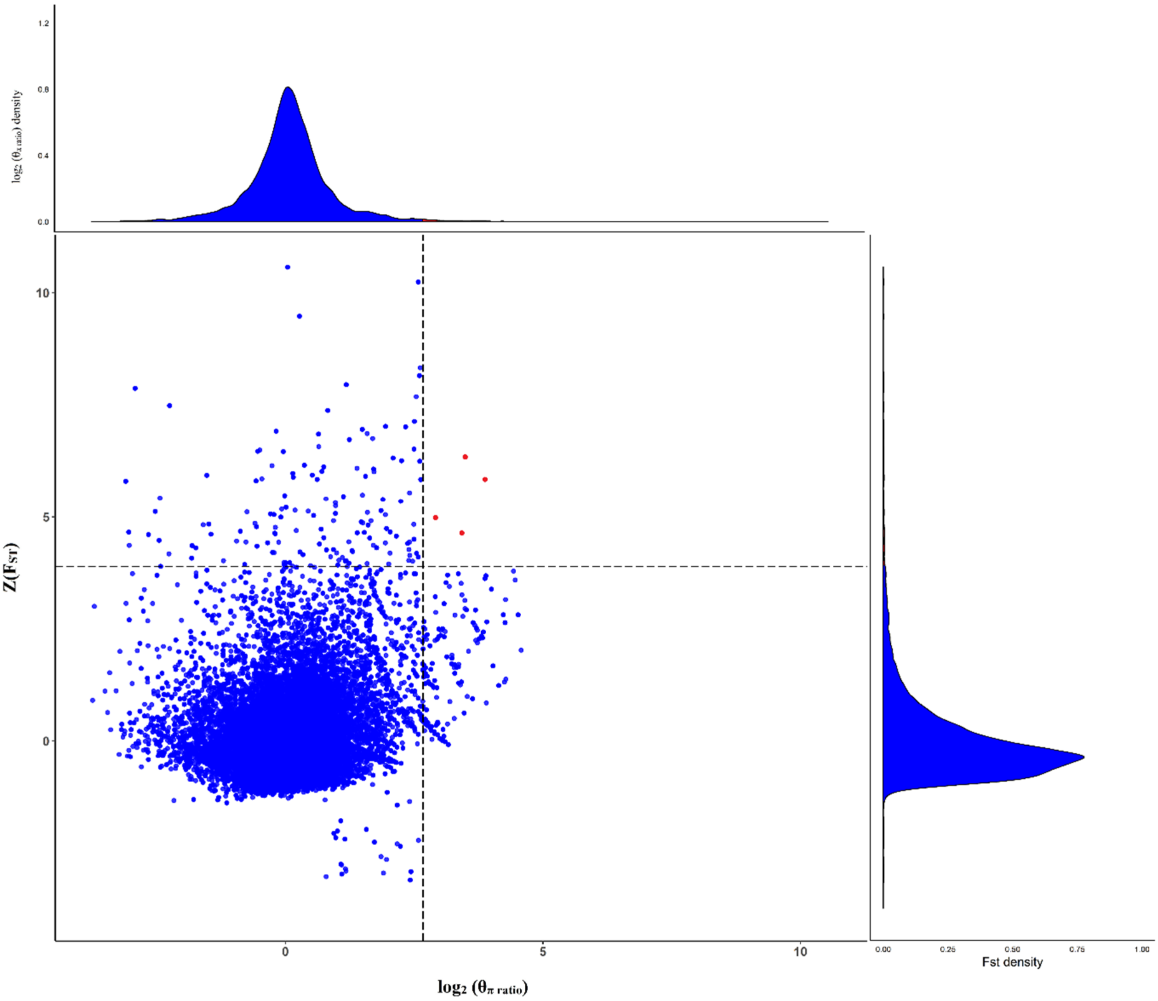

Supplementary Figure S7. Distribution of θπ ratios (θπ $_{Negative}$ / θπ $_{Positive}$) and Z(F_{ST}) values in antibody titer group. Red points correspond to the overlap of top 1% of empirical *log*$_2$ (θπ ratios) and Z(F_{ST}) distributions. The two horizontal and vertical dashed lines represent the top 1% value of Z(F_{ST}) (3.89) and *log*$_2$ (θπ ratios) (2.67), respectively. Only scaffolds >10 Mb were used for analyses.

Supplementary Table S1. The list of candidate regions along with their positions using both $Z(F_{ST})$ and log2 ($\theta_{\pi\ ratios}$) in different groups. Green color indicates the positions overlapped with regions detected by hapFLK test.

Group	Scaffolds	Start_point	End_point	Z(Fst)	log_2 ($\theta_{\pi\ ratio}$)
Survival	1	25275001	25375000	5.246026441	4.360714536
Survival	1	25300001	25400000	5.246026441	4.360714536
Survival	1	25325001	25425000	5.246026441	4.360714536
Survival	1	27700001	27800000	4.129817145	2.635642176
Survival	2	14250001	14350000	4.811266988	3.267257234
Survival	2	14275001	14375000	4.811266988	3.267257234
Survival	2	14300001	14400000	4.811266988	3.267257234
Survival	2	23425001	23525000	5.566964352	3.088257526
Survival	2	23450001	23550000	5.566964352	3.088257526
Survival	2	23475001	23575000	5.566964352	3.088257526
Survival	2	23500001	23600000	5.566964352	3.088257526
Survival	2	27800001	27900000	5.251967167	3.138145547
Survival	2	27825001	27925000	5.251967167	3.138145547
Survival	5	200001	300000	6.222917615	2.989058706
Survival	5	225001	325000	6.222917615	2.989058706
Survival	5	13850001	13950000	3.84614257	4.413492602
Survival	5	13875001	13975000	3.84614257	4.413492602
Survival	10	4875001	4975000	4.060747534	2.979597907
Survival	10	4900001	5000000	4.060747534	2.979597907
Survival	10	17425001	17525000	5.192975986	2.801045346
Kidney lesions	3	13850001	13950000	5.402726117	2.324715697
Kidney lesions	3	13875001	13975000	5.402726117	2.324715697
Kidney lesions	3	13900001	14000000	5.402726117	2.324715697
Kidney lesions	3	13925001	14025000	5.402726117	2.324715697
Kidney lesions	3	13950001	14050000	5.402726117	2.324715697
Kidney lesions	3	15500001	15600000	4.301597176	3.027463922
Kidney lesions	3	15525001	15625000	4.301597176	3.027463922
Kidney lesions	3	15550001	15650000	4.301597176	3.027463922
Kidney lesions	4	10700001	10800000	6.057667873	2.341248647
Kidney lesions	4	10725001	10825000	6.057667873	2.341248647
Kidney lesions	4	16300001	16400000	4.974008373	2.320539339
Kidney lesions	4	16325001	16425000	4.974008373	2.320539339
Kidney lesions	5	4125001	4225000	5.165273533	2.887445126
Kidney lesions	5	9450001	9550000	4.168836419	1.899164855
Kidney lesions	5	13850001	13950000	6.318735904	2.528779496
Kidney lesions	5	13875001	13975000	6.318735904	2.528779496
Kidney lesions	5	18625001	18725000	5.302822286	2.287016448
Kidney lesions	5	18650001	18750000	5.302822286	2.287016448
Kidney lesions	6	3050001	3150000	4.353760856	1.834393934
Kidney lesions	6	8150001	8250000	4.576440054	2.495123513
Kidney lesions	6	13825001	13925000	5.014643919	2.38891436
Kidney lesions	6	13850001	13950000	5.014643919	2.38891436
Kidney lesions	6	18375001	18475000	4.262883818	1.879939488
Kidney lesions	6	18400001	18500000	4.262883818	1.879939488
Kidney lesions	7	14400001	14500000	5.904242349	3.025871596
Kidney lesions	7	14425001	14525000	5.904242349	3.025871596
Kidney lesions	7	23700001	23800000	4.673293348	3.310265361
Kidney lesions	7	23725001	23825000	4.673293348	3.310265361

Kidney lesions	8	3625001	3725000	4.42860635	2.669498721
Kidney lesions	8	3650001	3750000	4.42860635	2.669498721
Kidney lesions	8	9400001	9500000	4.270857152	2.437334798
Kidney lesions	36	550001	650000	5.284998364	1.929941746
Kidney lesions	36	16800001	16900000	5.671233264	2.169253353
Kidney lesions	36	16825001	16925000	5.671233264	2.169253353
Viremia-350	1	17425001	17525000	4.982784932	3.075970385
Viremia-350	1	17450001	17550000	4.982784932	3.075970385
Viremia-350	1	17475001	17575000	4.982784932	3.075970385
Viremia-350	1	17500001	17600000	4.982784932	3.075970385
Viremia-350	2	24025001	24125000	4.0762644	2.69062622
Viremia-350	2	24050001	24150000	4.0762644	2.69062622
Viremia-350	2	27325001	27425000	5.208175186	2.972715655
Viremia-350	2	27350001	27450000	5.208175186	2.972715655
Viremia-350	3	1250001	1350000	4.641666815	4.131944479
Viremia-350	3	6650001	6750000	6.347741256	3.489639547
Viremia-350	4	5150001	5250000	4.140720863	2.063003229
Viremia-350	4	12725001	12825000	5.015600702	3.469813022
Virus clearance	1	4475001	4575000	5.17772993	3.120776155
Virus clearance	1	4500001	4600000	5.17772993	3.120776155
Virus clearance	1	4525001	4625000	5.17772993	3.120776155
Virus clearance	1	23825001	23925000	5.855722674	2.145024861
Virus clearance	1	23850001	23950000	5.855722674	2.145024861
Virus clearance	1	24525001	24625000	4.24049946	2.852644226
Virus clearance	1	24550001	24650000	4.24049946	2.852644226
Virus clearance	5	16525001	16625000	4.840459745	2.578178404
Virus clearance	5	22550001	22650000	6.213485785	2.848427929
Virus clearance	6	13475001	13575000	4.271039674	3.661194209
Virus clearance	6	13500001	13600000	4.271039674	3.661194209
Virus clearance	6	19295001	19395000	4.748617798	3.402891985
Virus clearance	6	19320001	19420000	4.748617798	3.402891985
Virus clearance	7	5000001	5100000	4.491991481	3.173571158
Virus clearance	7	5025001	5125000	4.491991481	3.173571158
Virus clearance	7	7325001	7425000	5.236154687	2.511437605
Virus clearance	7	19350001	19450000	5.430638308	2.185849833
Virus clearance	7	19375001	19475000	5.430638308	2.185849833
Virus clearance	7	19400001	19500000	5.430638308	2.185849833
Virus clearance	8	13950001	14025000	4.983910227	2.51297736
Virus clearance	8	21400001	21500000	6.804682356	3.161874181
Antibody titer	1	22800001	22900000	4.243799362	2.978676791
Antibody titer	1	22825001	22925000	4.243799362	2.978676791
Antibody titer	1	23000001	23100000	5.84574119	3.874499596
Antibody titer	1	23025001	23125000	5.84574119	3.874499596
Antibody titer	1	23050001	23150000	5.84574119	3.874499596
Antibody titer	1	23075001	23175000	5.84574119	3.874499596
Antibody titer	2	13750001	13850000	6.347741256	3.489639547
Antibody titer	2	13775001	13875000	6.347741256	3.489639547
Antibody titer	2	20375001	20475000	4.982784932	2.91149959
Antibody titer	2	20400001	20500000	4.982784932	2.91149959
Antibody titer	2	23425001	23525000	4.641666815	3.425424983
Antibody titer	2	23450001	23550000	4.641666815	3.425424983

Supplementary Table S2. The positions and Ensembl IDs of genes located in the putatively selected regions as well as their HGNC symbol and Ensembl Transcript ID.

Scaffolds	Start_position	End_position	Ensembl Gene ID	HGNC symbol	Ensembl Transcript ID
FNWR01000001.1	17450596	17592561	ENSNVIG00000006913	*PPP2R5E*	ENSNVIT00000010221
FNWR01000001.1	17450596	17592561	ENSNVIG00000006913	*PPP2R5E*	ENSNVIT00000010227
FNWR01000001.1	17450596	17592561	ENSNVIG00000006913	*PPP2R5E*	ENSNVIT00000010221
FNWR01000001.1	17450596	17592561	ENSNVIG00000006913	*PPP2R5E*	ENSNVIT00000010227
FNWR01000001.1	17450596	17592561	ENSNVIG00000006913	*PPP2R5E*	ENSNVIT00000010221
FNWR01000001.1	17450596	17592561	ENSNVIG00000006913	*PPP2R5E*	ENSNVIT00000010227
FNWR01000001.1	17450596	17592561	ENSNVIG00000006913	*PPP2R5E*	ENSNVIT00000010221
FNWR01000001.1	17450596	17592561	ENSNVIG00000006913	*PPP2R5E*	ENSNVIT00000010227
FNWR01000001.1	22800852	22862555	ENSNVIG00000009977	*SLC39A9*	ENSNVIT00000014773
FNWR01000001.1	22800852	22862555	ENSNVIG00000009977	*SLC39A9*	ENSNVIT00000014782
FNWR01000001.1	22876485	22905981	ENSNVIG00000010001	*PLEKHD1*	ENSNVIT00000014794
FNWR01000001.1	22800852	22862555	ENSNVIG00000009977	*SLC39A9*	ENSNVIT00000014773
FNWR01000001.1	22800852	22862555	ENSNVIG00000009977	*SLC39A9*	ENSNVIT00000014782
FNWR01000001.1	22876485	22905981	ENSNVIG00000010001	*PLEKHD1*	ENSNVIT00000014794
FNWR01000001.1	22977523	23070302	ENSNVIG00000010030	-	ENSNVIT00000014824
FNWR01000001.1	22977523	23070302	ENSNVIG00000010030	-	ENSNVIT00000014843
FNWR01000001.1	22977523	23070302	ENSNVIG00000010030	-	ENSNVIT00000014824
FNWR01000001.1	22977523	23070302	ENSNVIG00000010030	-	ENSNVIT00000014843
FNWR01000001.1	23114157	23118797	ENSNVIG00000010055	*SRSF5*	ENSNVIT00000014897
FNWR01000001.1	23114157	23118797	ENSNVIG00000010055	*SRSF5*	ENSNVIT00000014925
FNWR01000001.1	23114157	23118797	ENSNVIG00000010055	*SRSF5*	ENSNVIT00000014934
FNWR01000001.1	23122208	23155874	ENSNVIG00000010118	*SLC10A1*	ENSNVIT00000014984
FNWR01000001.1	22977523	23070302	ENSNVIG00000010030	-	ENSNVIT00000014824
FNWR01000001.1	22977523	23070302	ENSNVIG00000010030	-	ENSNVIT00000014843
FNWR01000001.1	23114157	23118797	ENSNVIG00000010055	*SRSF5*	ENSNVIT00000014897
FNWR01000001.1	23114157	23118797	ENSNVIG00000010055	*SRSF5*	ENSNVIT00000014925
FNWR01000001.1	23114157	23118797	ENSNVIG00000010055	*SRSF5*	ENSNVIT00000014934
FNWR01000001.1	23122208	23155874	ENSNVIG00000010118	*SLC10A1*	ENSNVIT00000014984
FNWR01000001.1	23114157	23118797	ENSNVIG00000010055	*SRSF5*	ENSNVIT00000014897
FNWR01000001.1	23114157	23118797	ENSNVIG00000010055	*SRSF5*	ENSNVIT00000014925
FNWR01000001.1	23114157	23118797	ENSNVIG00000010055	*SRSF5*	ENSNVIT00000014934
FNWR01000001.1	23122208	23155874	ENSNVIG00000010118	*SLC10A1*	ENSNVIT00000014984
FNWR01000001.1	23770263	23851983	ENSNVIG00000010373	*RIN3*	ENSNVIT00000015353
FNWR01000001.1	23908217	24072621	ENSNVIG00000010377	*SLC24A4*	ENSNVIT00000015447
FNWR01000001.1	23770263	23851983	ENSNVIG00000010373	*RIN3*	ENSNVIT00000015353
FNWR01000001.1	23908217	24072621	ENSNVIG00000010377	*SLC24A4*	ENSNVIT00000015447
FNWR01000001.1	24563396	24673404	ENSNVIG00000010959	*CATSPERB*	ENSNVIT00000016300
FNWR01000001.1	24572755	24579221	ENSNVIG00000010995	-	ENSNVIT00000016313

FNWR01000001.1	24563396	24673404	ENSNVIG00000010959	*CATSPERB*	ENSNVIT00000016300
FNWR01000001.1	24572755	24579221	ENSNVIG00000010995	-	ENSNVIT00000016313
FNWR01000001.1	25289075	25535573	ENSNVIG00000011336	*TTC7B*	ENSNVIT00000016886
FNWR01000001.1	25289075	25535573	ENSNVIG00000011336	*TTC7B*	ENSNVIT00000016886
FNWR01000001.1	25289075	25535573	ENSNVIG00000011336	*TTC7B*	ENSNVIT00000016886
FNWR01000001.1	27715978	27717000	ENSNVIG00000013328	*GPR65*	ENSNVIT00000019808
FNWR01000001.1	27742697	27800957	ENSNVIG00000013338	*GALC*	ENSNVIT00000019885
FNWR01000001.1	27742697	27800957	ENSNVIG00000013338	*GALC*	ENSNVIT00000019904
FNWR01000002.1	13849692	13927520	ENSNVIG00000020556	*GALNT1*	ENSNVIT00000030824
FNWR01000002.1	13849692	13927520	ENSNVIG00000020556	*GALNT1*	ENSNVIT00000030841
FNWR01000002.1	13849692	13927520	ENSNVIG00000020556	*GALNT1*	ENSNVIT00000030824
FNWR01000002.1	13849692	13927520	ENSNVIG00000020556	*GALNT1*	ENSNVIT00000030841
FNWR01000002.1	14255595	14268617	ENSNVIG00000020660	*SLC39A6*	ENSNVIT00000030961
FNWR01000002.1	14271675	14314014	ENSNVIG00000020672	*ELP2*	ENSNVIT00000031043
FNWR01000002.1	14271675	14314014	ENSNVIG00000020672	*ELP2*	ENSNVIT00000031052
FNWR01000002.1	14324814	14379730	ENSNVIG00000020726	*MOCOS*	ENSNVIT00000031150
FNWR01000002.1	14271675	14314014	ENSNVIG00000020672	*ELP2*	ENSNVIT00000031043
FNWR01000002.1	14271675	14314014	ENSNVIG00000020672	*ELP2*	ENSNVIT00000031052
FNWR01000002.1	14324814	14379730	ENSNVIG00000020726	*MOCOS*	ENSNVIT00000031150
FNWR01000002.1	14271675	14314014	ENSNVIG00000020672	*ELP2*	ENSNVIT00000031043
FNWR01000002.1	14271675	14314014	ENSNVIG00000020672	*ELP2*	ENSNVIT00000031052
FNWR01000002.1	14324814	14379730	ENSNVIG00000020726	*MOCOS*	ENSNVIT00000031150
FNWR01000002.1	14392468	14851673	ENSNVIG00000020821	*FHOD3*	ENSNVIT00000031444
FNWR01000002.1	14392468	14851673	ENSNVIG00000020821	*FHOD3*	ENSNVIT00000031446
FNWR01000002.1	14392468	14851673	ENSNVIG00000020821	*FHOD3*	ENSNVIT00000031449
FNWR01000002.1	14392468	14851673	ENSNVIG00000020821	*FHOD3*	ENSNVIT00000031464
FNWR01000002.1	20275708	20663251	ENSNVIG00000021238	*RIT2*	ENSNVIT00000031881
FNWR01000002.1	20275708	20663251	ENSNVIG00000021238	*RIT2*	ENSNVIT00000031894
FNWR01000002.1	20275708	20663251	ENSNVIG00000021238	*RIT2*	ENSNVIT00000031881
FNWR01000002.1	20275708	20663251	ENSNVIG00000021238	*RIT2*	ENSNVIT00000031894
FNWR01000002.1	23414563	23506149	ENSNVIG00000000779	*RNF165*	ENSNVIT00000001065
FNWR01000002.1	23519347	23670366	ENSNVIG00000000795	*LOXHD1*	ENSNVIT00000001460
FNWR01000002.1	23519347	23670366	ENSNVIG00000000795	*LOXHD1*	ENSNVIT00000001461
FNWR01000002.1	23519347	23670366	ENSNVIG00000000795	*LOXHD1*	ENSNVIT00000001462
FNWR01000002.1	23414563	23506149	ENSNVIG00000000779	*RNF165*	ENSNVIT00000001065
FNWR01000002.1	23519347	23670366	ENSNVIG00000000795	*LOXHD1*	ENSNVIT00000001460
FNWR01000002.1	23519347	23670366	ENSNVIG00000000795	*LOXHD1*	ENSNVIT00000001461
FNWR01000002.1	23519347	23670366	ENSNVIG00000000795	*LOXHD1*	ENSNVIT00000001462
FNWR01000002.1	23414563	23506149	ENSNVIG00000000779	*RNF165*	ENSNVIT00000001065
FNWR01000002.1	23519347	23670366	ENSNVIG00000000795	*LOXHD1*	ENSNVIT00000001460
FNWR01000002.1	23519347	23670366	ENSNVIG00000000795	*LOXHD1*	ENSNVIT00000001461

FNWR01000002.1	23519347	23670366	ENSNVIG00000000795	*LOXHD1*	ENSNVIT00000001462
FNWR01000002.1	23414563	23506149	ENSNVIG00000000779	*RNF165*	ENSNVIT00000001065
FNWR01000002.1	23519347	23670366	ENSNVIG00000000795	*LOXHD1*	ENSNVIT00000001460
FNWR01000002.1	23519347	23670366	ENSNVIG00000000795	*LOXHD1*	ENSNVIT00000001461
FNWR01000002.1	23519347	23670366	ENSNVIG00000000795	*LOXHD1*	ENSNVIT00000001462
FNWR01000002.1	23414563	23506149	ENSNVIG00000000779	*RNF165*	ENSNVIT00000001065
FNWR01000002.1	23519347	23670366	ENSNVIG00000000795	*LOXHD1*	ENSNVIT00000001460
FNWR01000002.1	23519347	23670366	ENSNVIG00000000795	*LOXHD1*	ENSNVIT00000001461
FNWR01000002.1	23519347	23670366	ENSNVIG00000000795	*LOXHD1*	ENSNVIT00000001462
FNWR01000002.1	23414563	23506149	ENSNVIG00000000779	*RNF165*	ENSNVIT00000001065
FNWR01000002.1	23519347	23670366	ENSNVIG00000000795	*LOXHD1*	ENSNVIT00000001460
FNWR01000002.1	23519347	23670366	ENSNVIG00000000795	*LOXHD1*	ENSNVIT00000001461
FNWR01000002.1	23519347	23670366	ENSNVIG00000000795	*LOXHD1*	ENSNVIT00000001462
FNWR01000002.1	24013898	24030040	ENSNVIG00000001270	-	ENSNVIT00000001817
FNWR01000002.1	24057352	24091387	ENSNVIG00000001287	*SKOR2*	ENSNVIT00000001851
FNWR01000002.1	24057352	24091387	ENSNVIG00000001287	*SKOR2*	ENSNVIT00000001851
FNWR01000003.1	1175701	1470315	ENSNVIG00000005676	*HYDIN*	ENSNVIT00000008976
FNWR01000003.1	13848188	13875385	ENSNVIG00000012297	*GOT2*	ENSNVIT00000018309
FNWR01000003.1	13880697	13893150	ENSNVIG00000012380	-	ENSNVIT00000018480
FNWR01000003.1	13880697	13893150	ENSNVIG00000012380	-	ENSNVIT00000018526
FNWR01000003.1	13947925	14030557	ENSNVIG00000012524	*CNOT1*	ENSNVIT00000019093
FNWR01000003.1	13947925	14030557	ENSNVIG00000012524	*CNOT1*	ENSNVIT00000019193
FNWR01000003.1	13848188	13875385	ENSNVIG00000012297	*GOT2*	ENSNVIT00000018309
FNWR01000003.1	13880697	13893150	ENSNVIG00000012380	-	ENSNVIT00000018480
FNWR01000003.1	13880697	13893150	ENSNVIG00000012380	-	ENSNVIT00000018526
FNWR01000003.1	13947925	14030557	ENSNVIG00000012524	*CNOT1*	ENSNVIT00000019093
FNWR01000003.1	13947925	14030557	ENSNVIG00000012524	*CNOT1*	ENSNVIT00000019193
FNWR01000003.1	13947925	14030557	ENSNVIG00000012524	*CNOT1*	ENSNVIT00000019093
FNWR01000003.1	13947925	14030557	ENSNVIG00000012524	*CNOT1*	ENSNVIT00000019193
FNWR01000003.1	13947925	14030557	ENSNVIG00000012524	*CNOT1*	ENSNVIT00000019093
FNWR01000003.1	13947925	14030557	ENSNVIG00000012524	*CNOT1*	ENSNVIT00000019193
FNWR01000003.1	13947925	14030557	ENSNVIG00000012524	*CNOT1*	ENSNVIT00000019093
FNWR01000003.1	13947925	14030557	ENSNVIG00000012524	*CNOT1*	ENSNVIT00000019193
FNWR01000003.1	14032403	14036007	ENSNVIG00000013007	*SETD6*	ENSNVIT00000019466
FNWR01000003.1	14039330	14077435	ENSNVIG00000013270	*NDRG4*	ENSNVIT00000020792
FNWR01000003.1	14039330	14077435	ENSNVIG00000013270	*NDRG4*	ENSNVIT00000020811
FNWR01000003.1	14039330	14077435	ENSNVIG00000013270	*NDRG4*	ENSNVIT00000020831
FNWR01000003.1	14039330	14077435	ENSNVIG00000013270	*NDRG4*	ENSNVIT00000020837
FNWR01000003.1	15517502	15547557	ENSNVIG00000016497	-	ENSNVIT00000024591
FNWR01000003.1	15517502	15547557	ENSNVIG00000016497	-	ENSNVIT00000024617
FNWR01000003.1	15517502	15547557	ENSNVIG00000016497	-	ENSNVIT00000024641

FNWR01000003.1	15550896	15578654	ENSNVIG00000016578	*OGFOD1*	ENSNVIT00000024670
FNWR01000003.1	15550896	15578654	ENSNVIG00000016578	*OGFOD1*	ENSNVIT00000024693
FNWR01000003.1	15550896	15578654	ENSNVIG00000016578	*OGFOD1*	ENSNVIT00000024695
FNWR01000003.1	15550896	15578654	ENSNVIG00000016578	*OGFOD1*	ENSNVIT00000024713
FNWR01000003.1	15578250	15594985	ENSNVIG00000016619	-	ENSNVIT00000024728
FNWR01000003.1	15517502	15547557	ENSNVIG00000016497	-	ENSNVIT00000024591
FNWR01000003.1	15517502	15547557	ENSNVIG00000016497	-	ENSNVIT00000024617
FNWR01000003.1	15517502	15547557	ENSNVIG00000016497	-	ENSNVIT00000024641
FNWR01000003.1	15550896	15578654	ENSNVIG00000016578	*OGFOD1*	ENSNVIT00000024670
FNWR01000003.1	15550896	15578654	ENSNVIG00000016578	*OGFOD1*	ENSNVIT00000024693
FNWR01000003.1	15550896	15578654	ENSNVIG00000016578	*OGFOD1*	ENSNVIT00000024695
FNWR01000003.1	15550896	15578654	ENSNVIG00000016578	*OGFOD1*	ENSNVIT00000024713
FNWR01000003.1	15578250	15594985	ENSNVIG00000016619	-	ENSNVIT00000024728
FNWR01000003.1	15601932	15641714	ENSNVIG00000016632	*AMFR*	ENSNVIT00000024756
FNWR01000003.1	15550896	15578654	ENSNVIG00000016578	*OGFOD1*	ENSNVIT00000024670
FNWR01000003.1	15550896	15578654	ENSNVIG00000016578	*OGFOD1*	ENSNVIT00000024693
FNWR01000003.1	15550896	15578654	ENSNVIG00000016578	*OGFOD1*	ENSNVIT00000024695
FNWR01000003.1	15550896	15578654	ENSNVIG00000016578	*OGFOD1*	ENSNVIT00000024713
FNWR01000003.1	15578250	15594985	ENSNVIG00000016619	-	ENSNVIT00000024728
FNWR01000003.1	15601932	15641714	ENSNVIG00000016632	*AMFR*	ENSNVIT00000024756
FNWR01000003.1	15647788	15812091	ENSNVIG00000016652	*GNAO1*	ENSNVIT00000024782
FNWR01000003.1	15647788	15812091	ENSNVIG00000016652	*GNAO1*	ENSNVIT00000024795
FNWR01000003.1	6647373	6652297	ENSNVIG00000010533	*EXOC3L1*	ENSNVIT00000015631
FNWR01000003.1	6652654	6660289	ENSNVIG00000010557	*KIAA0895L*	ENSNVIT00000015646
FNWR01000003.1	6660896	6664506	ENSNVIG00000010569	-	ENSNVIT00000015659
FNWR01000003.1	6660896	6664506	ENSNVIG00000010569	-	ENSNVIT00000015665
FNWR01000003.1	6665387	6670231	ENSNVIG00000010576	*HSF4*	ENSNVIT00000015717
FNWR01000003.1	6670823	6674563	ENSNVIG00000010608	-	ENSNVIT00000015734
FNWR01000003.1	6670823	6674563	ENSNVIG00000010608	-	ENSNVIT00000015744
FNWR01000003.1	6674615	6680359	ENSNVIG00000010625	-	ENSNVIT00000015786
FNWR01000003.1	6682666	6683880	ENSNVIG00000010650	*B3GNT9*	ENSNVIT00000015807
FNWR01000003.1	6684709	6713803	ENSNVIG00000010652	*C16orf70*	ENSNVIT00000015827
FNWR01000003.1	6720860	6789732	ENSNVIG00000010680	*CBFB*	ENSNVIT00000015870
FNWR01000003.1	6720860	6789732	ENSNVIG00000010680	*CBFB*	ENSNVIT00000015885
FNWR01000004.1	10677133	10777986	ENSNVIG00000022946	*UBE2H*	ENSNVIT00000034494
FNWR01000004.1	10677133	10777986	ENSNVIG00000022946	*UBE2H*	ENSNVIT00000034499
FNWR01000004.1	10677133	10777986	ENSNVIG00000022946	*UBE2H*	ENSNVIT00000034494
FNWR01000004.1	10677133	10777986	ENSNVIG00000022946	*UBE2H*	ENSNVIT00000034499
FNWR01000004.1	12777212	13503730	ENSNVIG00000000535	*GRM8*	ENSNVIT00000000713
FNWR01000004.1	16226344	16757034	ENSNVIG00000001278	*CADPS2*	ENSNVIT00000002159
FNWR01000004.1	16226344	16757034	ENSNVIG00000001278	*CADPS2*	ENSNVIT00000002172

FNWR01000004.1	16226344	16757034	ENSNVIG00000001278	*CADPS2*	ENSNVIT00000002159
FNWR01000004.1	16226344	16757034	ENSNVIG00000001278	*CADPS2*	ENSNVIT00000002172
FNWR01000005.1	13929210	13932384	ENSNVIG00000006567	-	ENSNVIT00000009685
FNWR01000005.1	13929210	13932384	ENSNVIG00000006567	-	ENSNVIT00000009685
FNWR01000005.1	13929210	13932384	ENSNVIG00000006567	-	ENSNVIT00000009685
FNWR01000005.1	13929210	13932384	ENSNVIG00000006567	-	ENSNVIT00000009685
FNWR01000005.1	16611094	16620950	ENSNVIG00000008114	*FBXO5*	ENSNVIT00000012001
FNWR01000005.1	18625760	18681836	ENSNVIG00000008632	*NOX3*	ENSNVIT00000012778
FNWR01000005.1	18625760	18681836	ENSNVIG00000008632	*NOX3*	ENSNVIT00000012778
FNWR01000005.1	22579270	22580247	ENSNVIG00000010425	*MAS1*	ENSNVIT00000015433
FNWR01000005.1	22647490	22742166	ENSNVIG00000010447	*IGF2R*	ENSNVIT00000015653
FNWR01000005.1	4021224	4138711	ENSNVIG00000003825	*PREP*	ENSNVIT00000005619
FNWR01000005.1	9513176	9554659	ENSNVIG00000005784	*TRAF3IP2*	ENSNVIT00000008517
FNWR01000006.1	13442030	13651019	ENSNVIG00000013630	*PHLPP1*	ENSNVIT00000020381
FNWR01000006.1	13442030	13651019	ENSNVIG00000013630	*PHLPP1*	ENSNVIT00000020409
FNWR01000006.1	13442030	13651019	ENSNVIG00000013630	*PHLPP1*	ENSNVIT00000020381
FNWR01000006.1	13442030	13651019	ENSNVIG00000013630	*PHLPP1*	ENSNVIT00000020409
FNWR01000006.1	13930857	13960087	ENSNVIG00000013795	*TNFRSF11A*	ENSNVIT00000020639
FNWR01000006.1	13930857	13960087	ENSNVIG00000013795	*TNFRSF11A*	ENSNVIT00000020649
FNWR01000006.1	13930857	13960087	ENSNVIG00000013795	*TNFRSF11A*	ENSNVIT00000020654
FNWR01000006.1	13930857	13960087	ENSNVIG00000013795	*TNFRSF11A*	ENSNVIT00000020662
FNWR01000006.1	13930857	13960087	ENSNVIG00000013795	*TNFRSF11A*	ENSNVIT00000020666
FNWR01000006.1	18164352	18529761	ENSNVIG00000016923	*WDR7*	ENSNVIT00000025357
FNWR01000006.1	18164352	18529761	ENSNVIG00000016923	*WDR7*	ENSNVIT00000025429
FNWR01000006.1	18164352	18529761	ENSNVIG00000016923	*WDR7*	ENSNVIT00000025462
FNWR01000006.1	18164352	18529761	ENSNVIG00000016923	*WDR7*	ENSNVIT00000025357
FNWR01000006.1	18164352	18529761	ENSNVIG00000016923	*WDR7*	ENSNVIT00000025429
FNWR01000006.1	18164352	18529761	ENSNVIG00000016923	*WDR7*	ENSNVIT00000025462
FNWR01000006.1	19401446	19796213	ENSNVIG00000017179	*TCF4*	ENSNVIT00000027769
FNWR01000006.1	19401446	19796213	ENSNVIG00000017179	*TCF4*	ENSNVIT00000027794
FNWR01000006.1	19401446	19796213	ENSNVIG00000017179	*TCF4*	ENSNVIT00000027809
FNWR01000006.1	19401446	19796213	ENSNVIG00000017179	*TCF4*	ENSNVIT00000027861
FNWR01000006.1	19401446	19796213	ENSNVIG00000017179	*TCF4*	ENSNVIT00000027882
FNWR01000006.1	19401446	19796213	ENSNVIG00000017179	*TCF4*	ENSNVIT00000027913
FNWR01000006.1	19401446	19796213	ENSNVIG00000017179	*TCF4*	ENSNVIT00000027924
FNWR01000006.1	19401446	19796213	ENSNVIG00000017179	*TCF4*	ENSNVIT00000027769
FNWR01000006.1	19401446	19796213	ENSNVIG00000017179	*TCF4*	ENSNVIT00000027794
FNWR01000006.1	19401446	19796213	ENSNVIG00000017179	*TCF4*	ENSNVIT00000027809
FNWR01000006.1	19401446	19796213	ENSNVIG00000017179	*TCF4*	ENSNVIT00000027861
FNWR01000006.1	19401446	19796213	ENSNVIG00000017179	*TCF4*	ENSNVIT00000027882
FNWR01000006.1	19401446	19796213	ENSNVIG00000017179	*TCF4*	ENSNVIT00000027913

FNWR01000006.1	19401446	19796213	ENSNVIG00000017179	*TCF4*	ENSNVIT00000027924
FNWR01000006.1	7883090	8255086	ENSNVIG00000012061	*DOK6*	ENSNVIT00000017957
FNWR01000007.1	14468616	14468828	ENSNVIG00000003391	-	ENSNVIT00000004926
FNWR01000007.1	14468616	14468828	ENSNVIG00000003391	-	ENSNVIT00000004926
FNWR01000007.1	14508413	14538600	ENSNVIG00000003411	*EED*	ENSNVIT00000005013
FNWR01000007.1	14508413	14538600	ENSNVIG00000003411	*EED*	ENSNVIT00000005065
FNWR01000007.1	14508413	14538600	ENSNVIG00000003411	*EED*	ENSNVIT00000005073
FNWR01000007.1	5001661	5022306	ENSNVIG00000000391	*WNT11*	ENSNVIT00000000494
FNWR01000007.1	5001661	5022306	ENSNVIG00000000391	*WNT11*	ENSNVIT00000000501
FNWR01000007.1	5001661	5022306	ENSNVIG00000000391	*WNT11*	ENSNVIT00000000507
FNWR01000007.1	5001661	5022306	ENSNVIG00000000391	*WNT11*	ENSNVIT00000000514
FNWR01000007.1	7325943	7714086	ENSNVIG00000001212	*TENM4*	ENSNVIT00000001767
FNWR01000008.1	13892035	13955296	ENSNVIG00000013543	-	ENSNVIT00000020324
FNWR01000008.1	13963535	14020056	ENSNVIG00000013664	*VSNL1*	ENSNVIT00000020355
FNWR01000008.1	3674636	3765375	ENSNVIG00000008727	*SULF2*	ENSNVIT00000012983
FNWR01000008.1	3674636	3765375	ENSNVIG00000008727	*SULF2*	ENSNVIT00000013025
FNWR01000008.1	3674636	3765375	ENSNVIG00000008727	*SULF2*	ENSNVIT00000012983
FNWR01000008.1	3674636	3765375	ENSNVIG00000008727	*SULF2*	ENSNVIT00000013025
FNWR01000010.1	17416314	17429981	ENSNVIG00000023629	*CATSPER4*	ENSNVIT00000035539
FNWR01000010.1	17450670	17476483	ENSNVIG00000023633	-	ENSNVIT00000035557
FNWR01000010.1	17479111	17480712	ENSNVIG00000023641	*SH3BGRL3*	ENSNVIT00000035559
FNWR01000010.1	17481433	17491441	ENSNVIG00000023643	*UBXN11*	ENSNVIT00000035566
FNWR01000010.1	17515069	17516403	ENSNVIG00000023648	-	ENSNVIT00000035571
FNWR01000010.1	17518099	17533842	ENSNVIG00000023650	-	ENSNVIT00000035579
FNWR01000010.1	17518099	17533842	ENSNVIG00000023650	-	ENSNVIT00000035582
FNWR01000010.1	4857803	4904905	ENSNVIG00000006615	*CLSTN1*	ENSNVIT00000009830
FNWR01000010.1	4857803	4904905	ENSNVIG00000006615	*CLSTN1*	ENSNVIT00000009841
FNWR01000010.1	4857803	4904905	ENSNVIG00000006615	*CLSTN1*	ENSNVIT00000009854
FNWR01000010.1	4960551	4987178	ENSNVIG00000006725	-	ENSNVIT00000009889
FNWR01000010.1	4960551	4987178	ENSNVIG00000006725	-	ENSNVIT00000009903
FNWR01000010.1	4857803	4904905	ENSNVIG00000006615	*CLSTN1*	ENSNVIT00000009830
FNWR01000010.1	4857803	4904905	ENSNVIG00000006615	*CLSTN1*	ENSNVIT00000009841
FNWR01000010.1	4857803	4904905	ENSNVIG00000006615	*CLSTN1*	ENSNVIT00000009854
FNWR01000010.1	4960551	4987178	ENSNVIG00000006725	-	ENSNVIT00000009889
FNWR01000010.1	4960551	4987178	ENSNVIG00000006725	-	ENSNVIT00000009903
FNWR01000036.1	16341208	16833227	ENSNVIG00000010682	*SBF2*	ENSNVIT00000015893
FNWR01000036.1	16855353	16922589	ENSNVIG00000010729	*SWAP70*	ENSNVIT00000015947
FNWR01000036.1	16855353	16922589	ENSNVIG00000010729	*SWAP70*	ENSNVIT00000015963
FNWR01000036.1	16341208	16833227	ENSNVIG00000010682	*SBF2*	ENSNVIT00000015893
FNWR01000036.1	16855353	16922589	ENSNVIG00000010729	*SWAP70*	ENSNVIT00000015947
FNWR01000036.1	16855353	16922589	ENSNVIG00000010729	*SWAP70*	ENSNVIT00000015963

FNWR01000036.1	522976	724785	ENSNVIG00000004371	*GFRA1*	ENSNVIT00000006459
FNWR01000036.1	522976	724785	ENSNVIG00000004371	*GFRA1*	ENSNVIT00000006469
